# Lattice Carbon‐Mediated Ultralow‐Barrier C–C Coupling for Selective CO Electroreduction to Ethylene

**DOI:** 10.1002/advs.202521983

**Published:** 2025-12-23

**Authors:** Jiangke Tao, Zhichao Yu, Lulu Chen, Weng Fai Ip, Sen Lin, Hui Pan

**Affiliations:** ^1^ Institute of Applied Physics and Materials Engineering University of Macau Macao SAR P. R. China; ^2^ State Key Laboratory of Photocatalysis on Energy and Environment College of Chemistry Fuzhou University Fuzhou P. R. China; ^3^ State Key Laboratory of Chemistry for NBC Hazards Protection College of Chemistry Fuzhou University Fuzhou P. R. China; ^4^ Department of Physics and Chemistry Faculty of Science and Technology University of Macau Macao SAR P. R. China

**Keywords:** AIMD, C─C coupling, CO reduction, DFT, lattice carbon

## Abstract

Understanding the mechanistic pathways of catalytic CO_2_ reduction is essential for the rational design of high‐performance electrocatalysts. A key challenge in converting CO_2_ to multi‐carbon (C_2+_) products is the high energy barrier for C─C coupling, which limits both activity and selectivity. Here, using combined density functional theory (DFT) and molecular dynamics simulations, we demonstrate that lattice carbon sites at the edges of MXene (Ti_2_C(OH)_2_) serve as highly effective adsorption centers for ^*^CO intermediates during CO electroreduction. Remarkably, these sites significantly reduce the C─C coupling barrier through a lattice carbon‐mediated mechanism (LCMM). Electronic structure analyses, including projected densities of states, Bader charge partitioning, differential charge density, and electron localization function calculations, reveal that the LCMM facilitates substantial electron transfer to adsorbed CO. This electron enrichment weakens the C≡O bond while simultaneously promoting C─C bond formation, overcoming conventional coupling limitations. Our findings provide fundamental insights into C─C bond formation mechanisms and establish new design principles for developing selective C_2_ electrocatalysts.

## Graphical abstract

Combined DFT and molecular dynamics simulations reveal that while the lattice carbon at the MXene edge cannot directly bind CO_2_ to form ethylene, it acts as an active center to promote C─C coupling between adjacent ^*^CO intermediates for ethylene production.

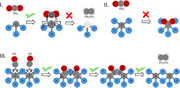

## 1 Introduction

The electrocatalytic CO_2_ reduction reaction (CO_2_RR) represents a highly promising strategy to address energy and environmental challenges associated with CO_2_ emission [1, 2]. This process enables the conversion of CO_2_ and H_2_O into valuable fuels and chemicals under mild conditions, offering precise control over reaction rates and product selectivity [3]. Over the past few decades, significant efforts have been devoted to developing efficient catalysts capable of achieving high activity and selectivity for CO_2_ conversion [4]. However, the catalytic performance—particularly for the production of multi‐carbon (C_2+_) products—remains unsatisfactory due to sluggish multi‐electron transfer kinetics and unresolved reaction pathways [5].

The activation of CO_2_ and subsequent C─C coupling are widely recognized as the critical steps governing the electrocatalytic conversion of CO_2_ to multi‐carbon (C_2+_) products. To date, most catalyst designs have focused on optimizing CO_2_ adsorption [6, 7] and stabilizing key intermediates [8, 9, 10, 11] to facilitate C─C coupling. However, these strategies often overlook the potential participation of lattice atoms from the catalyst itself—a mechanistic pathway that has proven highly effective in other electrochemical processes. Indeed, lattice‐atom participation has been demonstrated to enhance reaction kinetics across diverse systems, including the oxygen evolution reaction (OER) [12], nitrogen reduction reaction (NRR) [13, 14], and anionic redox reactions in lithium‐ion batteries [15]. This raises a compelling question: could an analogous lattice‐involved mechanism operate during CO_2_RR, particularly for C_2+_ formation? Such a pathway could provide dual advantages—lowering activation barriers of CO_2_ while simultaneously promoting C─C coupling—potentially opening new avenues for designing high‐performance CO_2_RR electrocatalysts.

Electrocatalysts exhibiting lattice‐involved mechanisms typically share elemental composition with their reactants. By analogy, effective CO_2_RR catalysts leveraging lattice participation should inherently contain carbon atoms. While experimental studies have demonstrated that lattice carbonates can enhance CO_2_ activation and C─C coupling during ethylene (C_2_H_4_) production with improved Faraday efficiency, systematic exploration of lattice carbon‐mediated CO_2_ reduction remains scarce [16]. To directly probe the mechanism of lattice‐carbon‐mediated CO_2_ reduction, an ideal model catalyst must possess high conductivity and, more importantly, exposed lattice carbon atoms. 2D MXenes meet these criteria perfectly: their metallic conductivity ensures rapid electron transport [17], while their atomic‐scale edges inherently expose lattice carbon sites adjacent to unsaturated metal centers [18]. Moreover, Ti_2_C(OH)_2_ has been theoretically predicted to deliver exceptionally low limiting potentials and high selectivity toward formic acid or formate by utilizing surface ‐OH as built‐in proton sources, outperforming many conventional metal electrocatalysts [19]. Recent high‐profile studies on Ti‐based MXenes, including single‐atom‐confined systems [20, 21] and comprehensive reviews [22], further underscore the extraordinary potential of this materials family for CO_2_RR. Therefore, we selected bilayer Ti_2_C(OH)_2_ MXene as the primary model system in this work to elucidate the intrinsic role of lattice carbon in mediating CO_2_ activation and multi‐carbon product formation.

Using Ti_2_C(OH)_2_ as a representative example, we first employed density‐functional theory (DFT) to evaluate the edge energies of various configurations, followed by ab‐initio molecular dynamics (AIMD) simulations to assess structural stability. Our calculations identified two low‐energy, thermodynamically stable edge structures with exposed carbon atoms. This work primarily addressed two questions: whether the lattice carbon atoms at these MXene edges can participate as a reactive carbon source, and how effectively these edges catalyze CO reduction to C_2_ products. The results reveal that although lattice carbon atoms remain anchored at the edges and do not directly incorporate into CO_2_ to form C_2_ products, they play a pivotal role in mediating ^*^CO coupling to generate ethylene (C_2_H_4_). Strikingly, one edge structure of Ti_2_C(OH)_2_ exhibits a near‐negligible energy barrier for ^*^CO‐^*^CO coupling (0.10 eV). Projected densities of states (PDOSs), Bader charge analysis, differential charge density mapping, and electron localization function (ELF) calculations collectively demonstrate that the electron‐rich nature of lattice carbons facilitates the C─C bond formation, significantly reducing the coupling barrier.

## 2 Computational Details

The bare M_2_C‐type MXenes exhibit a trilayered structure consisting of M─C─M atomic layers arranged in a face‐centered cubic (fcc) close‐packed configuration [23]. As MXenes are typically synthesized through HF etching of MAX phases, their surfaces are invariably functionalized with F, O, and/or OH groups [23]. We first evaluated the stability of MXenes with O, F, and OH functional groups in both vacuum and solvent environments. The calculated results confirm that all three terminations are thermodynamically stable (Figures S1 and S2). Based on this, our catalytic study encompasses all three structures, with Ti_2_C(OH)_2_ serving as the primary model system to investigate the reaction mechanisms at the MXene edges. The influence of the functional group on the catalytic performance is also discussed in this work.

**FIGURE 1:**
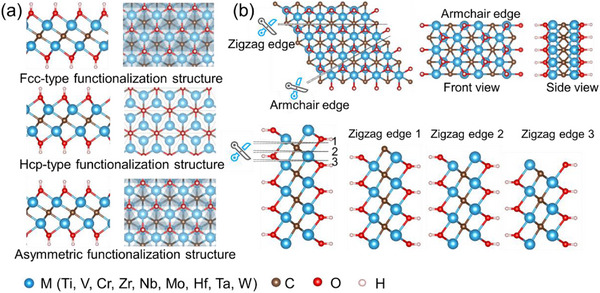
(a) Three functionalization structures for the M_2_C(OH)_2_ (M = Ti, V, Cr, Zr, Nb, Mo, Hf, Ta, W). (b) Four edge structures of Ti_2_C(OH)_2_ with the Fcc‐type functionalization structure shown as an example.

Based on Ti_2_C(OH)_2_, we constructed three distinct structural models (Figure 1a) [23]:

1. Fcc‐type functionalization structure: OH groups occupy face‐centered cubic (fcc) hollow sites on both surfaces with a monolayer (ML) coverage, maintaining fcc stacking symmetry.
2. Hcp‐type functionalization structure: OH groups adsorb at hexagonal close‐packed (hcp) hollow sites on both surfaces (a ML coverage), preserving hcp stacking.
3. Asymmetric functionalization structure: OH groups adopt fcc‐type adsorption on one surface and hcp‐type on another surface.

To identify the thermodynamically preferred functionalization structure for each transition metal (M), we performed full structural optimizations across the MXene series. The calculated total energies (Table S1) reveal that Fcc‐type functionalization structure is most stable for Ti, V, Zr, and Nb systems, Hcp‐type functionalization structure is energetically favored for Mo, Ta, and W systems, and the asymmetric functionalization structure is the ground state for Cr and Hf systems.

The edge structures of MXenes were generated by cleaving a MXene sheet along specific crystallographic directions (Figure 1b). Using Ti_2_C(OH)_2_ nanoribbons as a representative example, we identify two fundamental edge configurations based on their edge termination geometry: armchair edge (AC) and zigzag edge (ZZ). The zigzag configuration can be further classified into three distinct types (Zigzag edge 1—ZZ1, Zigzag edge 2—ZZ2, and Zigzag edge 3—ZZ3) depending on the precise cleavage position relative to the atomic lattice (Figure 1b). The edge structures of Hcp‐type functionalized and Asymmetric‐ functionalized MXenes are shown in Figure S3a,b. To quantitatively assess the thermodynamic stability of these edge structures, we calculated their edge formation energies using: [24]

(1) Eedge=12LEedgeunrelaxed−nEunit(Ti2C(OH)2)−mμTi−lμC−kμH−hμOEedgeunrelaxed+1LEedgerelaxed−Eedgeunrelaxed

$E_{edge}^{relaxed}$ is the total energy of nanoribbon after the structure relaxation. $E_{edge}^{unrelaxed}$ represents the total energy of nanoribbon at the initial atomic configuration (prior to any structural relaxation), corresponding to the first ionic step in the geometry optimization procedure. *E_unit_* (Ti_2_C(OH)_2_) is the energy of Ti_2_C(OH)_2_ unit cell. µ_*Ti*_, µ_*C*_, µ_*H*_, and µ_*O*_ are the chemical potentials of Ti, C, H, and O, and calculated from their bulk counterparts (Ti metal, graphene, hydrogen gas and oxygen gas). n is the total number of Ti_2_C(OH)_2_ units in the nanoribbon. *m, l, k*, and *h* are the numbers of extra Ti, C, H, and O atoms at the edges after deducting the *n*Ti_2_C(OH)_2_ units from the nanoribbon. L is the length of the nanoribbon. Note that the upper and lower edges of the nanoribbon must maintain stoichiometric complementarity, since they originate from a single cleavage of the pristine monolayer (i.e., the removed edge atoms at one boundary reappear at the opposite boundary, as shown in Figure S4a). This ensures proper accounting of the edge formation energy in Equation (2). The edge energies are presented in Table 1.

**TABLE 1:** The formation energy of different Ti_2_C(OH)_2_ edge types.

Ti_2_C(OH)_2_ edge types	E_edge_ (eV/Å)	Width of nanoribbon (Å)	Width of relaxed atomic layer (Å)
ZZ1	0.74	6.12	2.50
ZZ2	1.13	7.00	2.625
ZZ3	0.74	6.12	2.625
AC	1.14	6.06	1.56

All spin‐polarized DFT calculations, Born–Oppenheimer molecular dynamics (BOMD) simulations, and constrained MD simulations were performed using the Vienna ab initio Simulation Package (VASP) [25, 26, 27, 28, 29, 30, 31]. The Perdew‐Burke‐Ernzerhof (PBE) exchange‐correlation functional within the generalized gradient approximation (GGA) was employed [26, 32], with van der Waals corrections incorporated via the DFT‐D3 method [33]. Core‐electron interactions were treated using the projector augmented‐wave (PAW) method [34], while valence electrons were expanded in a plane‐wave basis set with a kinetic energy cutoff of 450 eV [30]. A dipole correction along the z‐direction was applied to account for the structural asymmetry between the upper and lower edges. The transition state and minimum energy paths were implemented using the climbing image nudged elastic band method (CI‐NEB) [35]. Structural optimizations were conducted using the Broyden–Fletcher–Goldfarb–Shanno (BFGS) algorithm until all atomic forces were below 0.02 eV/Å. Electronic convergence criteria were set to 10^−5^ eV for both total energy and electronic gradients [25, 30, 34]. A Gaussian smearing (ISMEAR = 0) of σ =  0.05 eV was applied throughout all calculations. Brillouin zone sampling utilized a 1 × 4 × 1 Monkhorst–Pack k‐point mesh for static DFT calculations and a Γ‐centered mesh for molecular dynamics simulations [36]. To eliminate periodic boundary interactions, a vacuum layer of 10.0 Å was introduced along *z*‐direction (see Figure S4a). Solvent effects were modeled by explicitly including 20 water molecules above the catalyst edge, with an additional 10 Å vacuum layer to isolate periodic images (see Figure S4b). Molecular dynamics simulations were performed in the canonical (NVT) ensemble using Nose‐Hoover thermostats at 300 K [37, 38]. We note the reported issue regarding temperature gradients with the Nose‐Hoover thermostat [39]. However, there is no significant gradient developed in our system (Figure S5), which supports the validity of our approach. Each trajectory was propagated for 5–10 ps with a 1.0 fs timestep. To enable this timestep, hydrogen atoms were assigned a mass of 2.0 am u (deuterium mass) in AIMD calculations. The crystal orbital Hamilton population (COHP) analysis was performed using the LOBSTER package [40].

Following a survey of recent theoretical studies on MXene‐based catalysts where the PBE functional has been predominantly employed [41, 42, 43], we also adopted this standard approach. To perform a robustness check on the potential influence of d‐state localization, we conducted supplementary DFT+U calculations on key reaction intermediates and barriers, as detailed in the Supporting Information (Figures S29, S30, and Table S3).

To quantitatively evaluate reaction thermodynamics and kinetics, including key processes such as ^*^CO coupling, intermediate hydrogenation, and C─C bond cleavage, we computed free energy profiles using thermodynamic integration (TI). This approach employs constrained molecular dynamics simulations, where the reaction coordinate (ζ) is systematically varied while applying holonomic constraints. The free energy change (ΔG) and kinetic barriers were determined by integrating the constraint force F(ζ) along ζ: [33, 44]

(2) $$\Delta G\left(\zeta_{a},\zeta_{b}\right)=-\int_{\zeta_{a}}^{\zeta_{b}}F\left(\zeta \right)d\zeta$$

where Δ*G*(ζ_*a*_,ζ_*b*_) is the free energy difference between two reaction coordinates (ζ_*a*_ and ζ_*b*_) and *F*(ζ) is the constrained force.

The free energy diagrams of proton‐electron transfer steps were modeled as a function of applied electrode potential (U) using the computational hydrogen electrode (CHE) framework developed by Nørskov and coworkers [45]. In this approach, the free energy change for adding a proton‐electron pair (H⁺ + e^−^) is calculated by referencing to the chemical potential of ½ H_2_ (gas) under standard conditions [U = 0 V vs. RHE (reversible hydrogen electrode), P(H_2_) = 1 atm, T = 298 K], as described by:

(3) $$\mu \left(H^{+}+e^{-}\right)=\frac{1}{2}\mu \left(H_{2}\right)-eU$$

where e is the electron charge, and U represents the applied electrode potential relative to RHE. For each elementary step, the change in the Gibbs reaction free energy (Δ*G*) can be expressed as:

(4) $$\Delta G=\Delta E+\Delta E_{ZPE}-T\Delta S+\Delta H$$

where Δ*E* is the total energy change; Δ*E_ZPE_* and Δ*S* are the change in zero‐point energy and the difference in entropy, respectively; Δ*H* is the variation of enthalpy; and T is the temperature (298K). The part of Δ*E_ZPE_* − *T*Δ*S* + Δ*H* were obtained from the vibrational frequency calculations through the VASPKIT code [46].

## 3 Results and Discussion

### 3.1 Stability and Electronic Structure Analysis of Ti_2_C(OH)_2_ Edges

**FIGURE 2:**
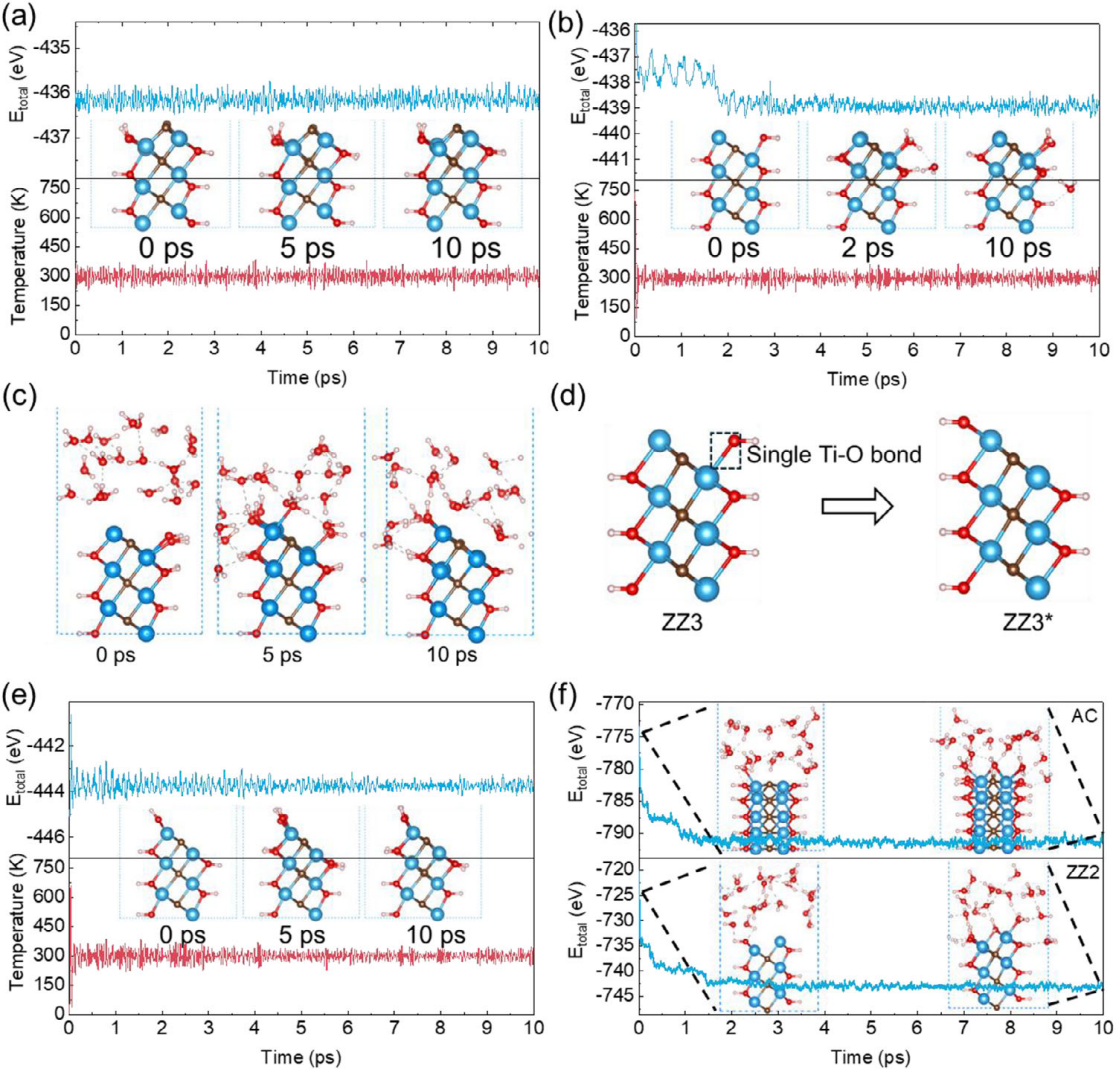
Evolution of total energy and temperature over time for (a) ZZ1 and (b) ZZ3. (c) Structural evolution of ZZ3 in solvent environment over 10 ps, demonstrating OH group detachment and edge reconstruction. (d) ZZ3 after structural modification, denoted as zigzag edge 3^*^ (ZZ3^*^). (e) Evolution of total energy and temperature over time for ZZ3^*^. (f) Atomic configuration changes of AC and ZZ2 structures in explicit aqueous solvent from AIMD simulations at 300 K. Insets show corresponding edge structures at selected time points.

Using Ti_2_C(OH)_2_ as a representative example, we systematically evaluated the stability and electronic properties of MXene edge structures. Catalyst stability represents a fundamental prerequisite for electrocatalytic CO_2_ reduction. Our calculations reveal that ZZ1 and ZZ3 exhibit lower edge formation energies (*E_edge_*) than ZZ2 and AC (Table 1). To assess dynamic stability, we performed AIMD simulations at 300 K for 10 ps. ZZ1 maintains structural integrity throughout the simulation (Figure 2a). In contrast, ZZ3 undergoes significant energy fluctuations during the initial 2 ps (Figure 2b), where a terminal OH group desorbs via proton transfer from a neighboring OH group, forming H_2_O. This suggests that singly coordinated terminal OH groups (Ti─O bond) are susceptible to reduction and desorption under operational conditions. To further investigate this behavior, we conducted an explicit‐solvent AIMD simulations of ZZ3. As predicted, the terminal OH groups undergo hydrogenation and detachment (Figure 2c), resulting in a modified edge structure (denoted ZZ3^*^), where the top Ti layer becomes fully OH‐terminated (Figure 2d). The ZZ3^*^ configuration shows excellent stability throughout the simulation (Figure 2e). Extending this analysis, we propose that ZZ2 and AC edges, which similarly feature unsaturated metal atoms, would also undergo spontaneous functionalization under reaction conditions. This hypothesis is confirmed by explicit‐solvent simulations (Figure 2f), showing rapid edge functionalization. Consequently, despite their initial exposure, these edge structures are unlikely to maintain catalytically active sites for CO_2_ reduction due to spontaneous passivation.

We performed the simulations on the stability of F‐ and O‐terminated Ti‐based MXenes, too. In a solvent environment, the AC and ZZ2 of Ti_2_CF_2_ are prone to re‐functionalization (Figure S6a,c), whereas the ZZ1 and ZZ3 remain stable (Figure S6b,d). Similarly, the AC and ZZ2 edges of Ti_2_CO_2_ also undergo re‐functionalization (Figure S7a,c). In contrast, the ZZ1 of Ti_2_CO_2_ is unstable, with its lattice carbon atoms are easily occupied by H and OH groups (Figures S7b and S8). The ZZ3 edge is structurally stable. However, its terminal oxygen atoms can be reduced to water molecules, and the exposed Ti atoms are susceptible to OH occupation (Figure S7d), which is analogous to the ZZ3^*^ structure of Ti_2_C(OH)_2_.

**FIGURE 3:**
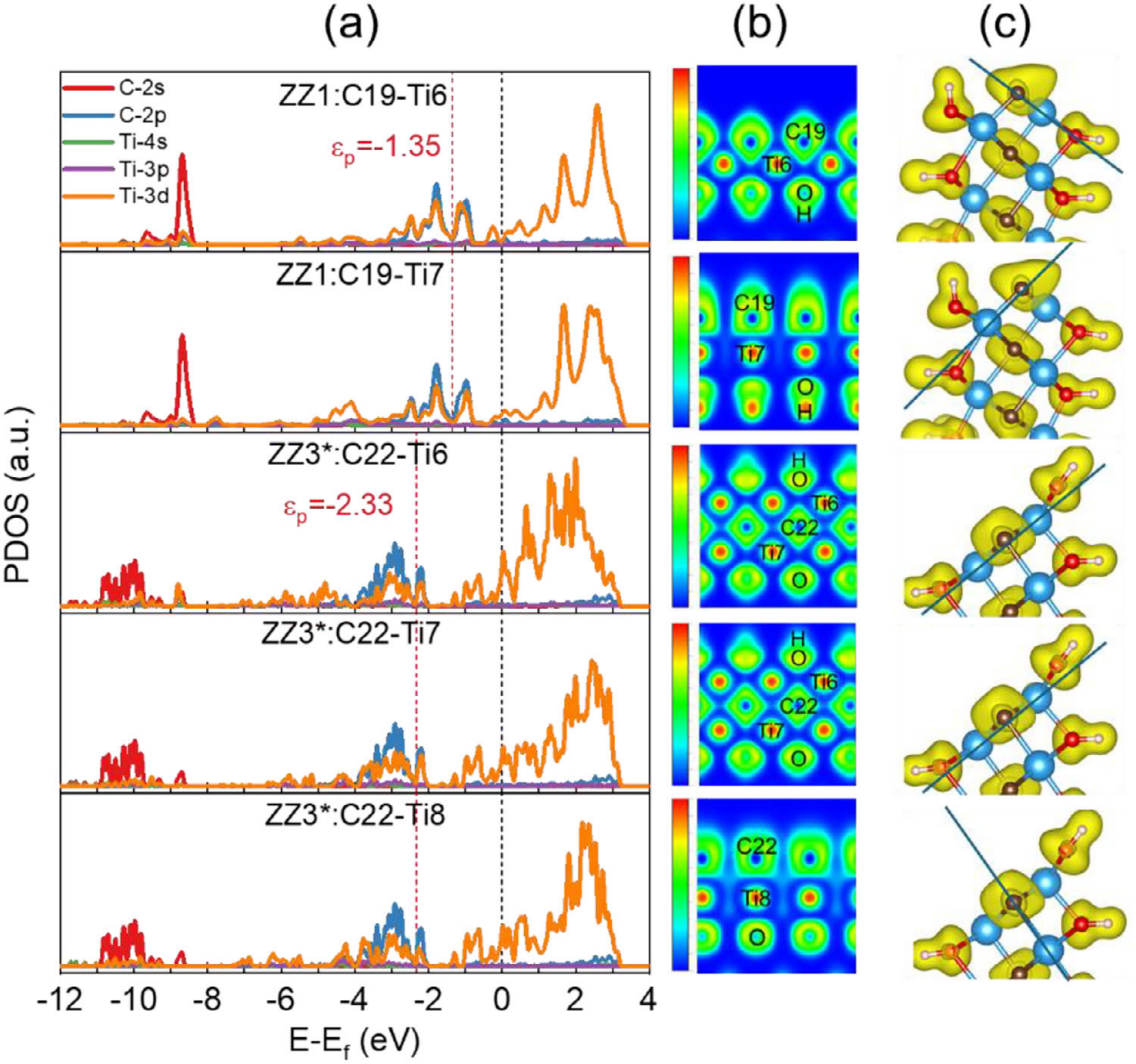
Electronic structures of active edge sites. (a) PDOSs for lattice carbon atoms and their coordinated Ti atoms at ZZ1 and ZZ3^*^. ε_*p*_ is the p‐band center of lattice carbon. (b) Cross‐sectional ELF plots through the active carbon sites. (c) ELF 3D diagram with the isosurface level at 0.45. The straight line shows the direction of cross‐section of Figure 3b.

Based on the calculated edge energy and dynamic stability, ZZ1 and ZZ3^*^ emerge as promising catalytic platforms. We systematically investigated their electronic properties through PDOSs and ELF analyses. PDOSs analysis reveals significant Ti‐3d/C‐2p orbital hybridization at both edges, with the p‐band center of lattice carbon (^*^C_lat_) atoms in ZZ1 located closer to the Fermi level (E_F_) (−1.35 eV) compared to that in ZZ3^*^ (−2.33 eV), suggesting enhanced electronic accessibility (Figure 3a). ELF calculations confirm polar covalent Ti─C bonding (Figure 3b), with electron density preferentially localized around the more electronegative carbon atoms (χ_*C*_ = 2.55 vs. χ_*Ti*_ = 1.54) and OH groups. Notably, ZZ1 exhibits higher electron delocalization around its lattice carbon atoms due to their reduced coordination by Ti atoms (3 for ZZ1 vs. 5 for ZZ3^*^ in average), as evidenced by both cross‐sectional and 3D ELF visualizations (Figure 3b,c). These electronic structure differences likely contribute to the distinct catalytic behaviors of these edge configurations.

### 3.2 Can Lattice Carbon Couple with CO_2_ and Desorb as C_2_H_4_?

**FIGURE 4:**
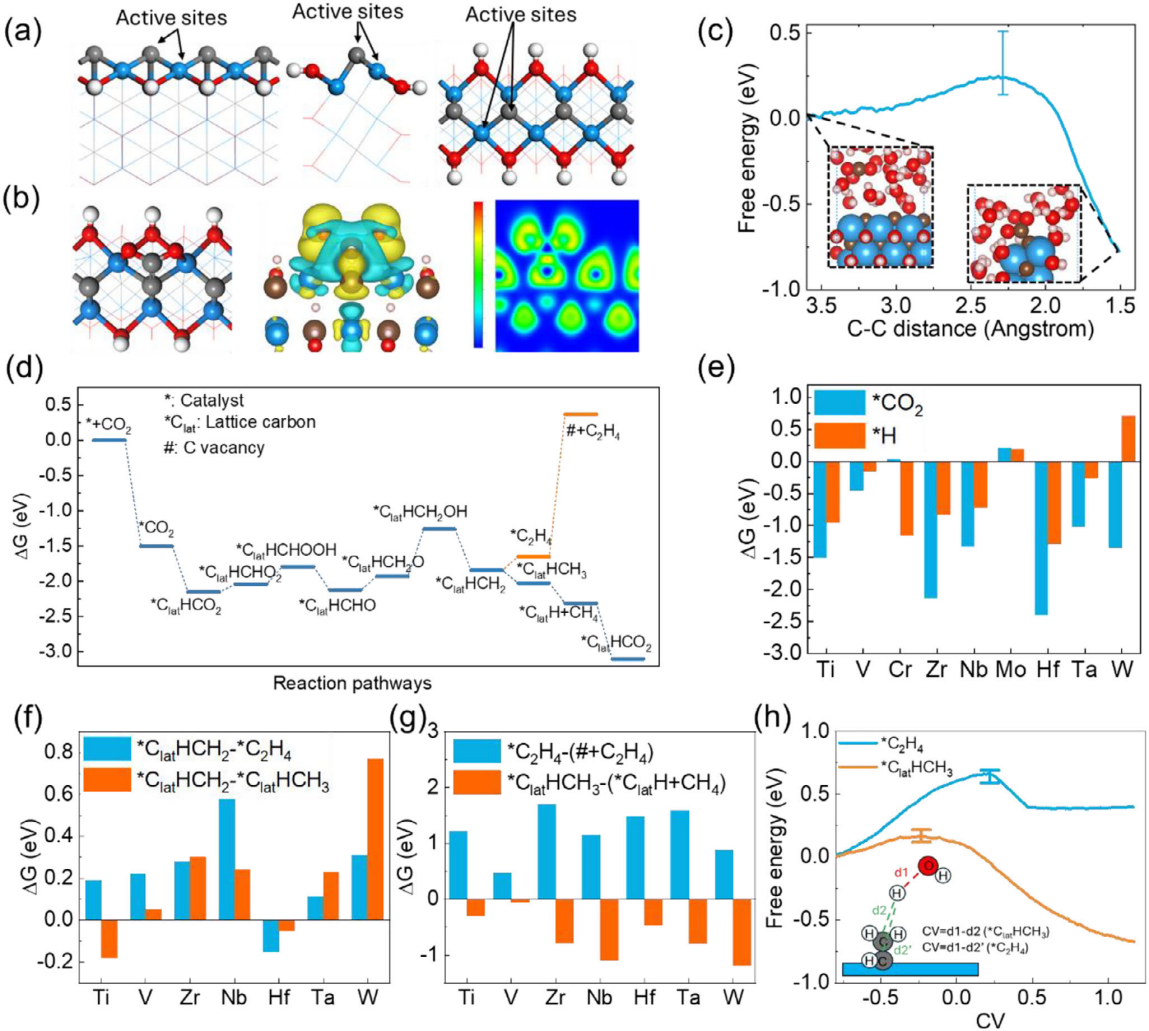
Structural and mechanistic analyses of CO_2_ reduction on MXene ZZ1. (a) Atomic structure of Ti_2_C(OH)_2_ ZZ1 shown in front, left, and top views. (b) CO_2_ adsorption configuration with corresponding differential charge density (isosurface = ±0.003 eÅ^−3^, yellow/blue for accumulation/depletion) and ELF analysis. (c) Energy profile for CO_2_ activation at ^*^C_lat_ with inset showing initio and final state configuration. (d) Proposed CO_2_ reduction pathways at 0 V vs. RHE. ^*^ means catalyst edge, # means C vacancy, and ^*^C_lat_ means lattice carbon. (e) Comparative adsorption energies of H and CO_2_ at ZZ1 across different metal carbides. (f) Gibbs free energy changes for ^*^C_lat_HCH_2_ hydrogenation to ^*^C_lat_HCH_3_ (C_1_ path) or ^*^C_2_H_4_ (C_2_ path). (g) Free energy comparison between C_2_H_4_ desorption and CH_4_ formation steps. (h) Kinetic barriers for competing hydrogenation pathways of ^*^C_lat_HCH_2_. Blue line is ^*^C_lat_HCH_2_→^*^C_2_H_4_ and orange line is ^*^C_lat_HCH_2_→^*^C_lat_HCH_3_. CV is equal to (d1–d2) and the reaction coordinate.

The ZZ1 edge structure was investigated first because the ^*^C_lat_ at ZZ1 edges exhibits lower metal coordination (3‐Ti) (Figure 4a) compared to ZZ3^*^ edges (5‐Ti), which can enhance both the reactivity of ^*^C_lat_ toward CO_2_ binding (higher ε_*p*_ of ^*^C_lat_) and the subsequent desorption of C_2_ product. This rationale is further supported by the trend that the formation energy of a carbon vacancy decreases as the number of its coordinating Ti─C bonds is reduced (Figure S9). Our analysis of Ti_2_C(OH)_2_ identifies CO_2_ adsorption configuration on the ZZ1: the carbon of CO_2_ binds to ^*^C_lat_ while its oxygen atoms coordinate to Ti sites (Figure 4b). Differential charge density analysis confirms the bonding between ^*^C_lat_ and the adsorbed CO_2_ carbon. ELF analysis confirms the covalent bonding between them. The low activation barrier of 0.25 eV (Figure 4c) demonstrates that CO_2_ readily couples to ^*^C_lat_ on the ZZ1 of Ti_2_C(OH)_2_.

Despite this favorable initial adsorption, subsequent reduction predominantly yields CH_4_ rather than C_2_ products (Figure 4d). In addition, we extended our investigation to other early transition metal carbides (V, Cr, Zr, Nb, Mo, Hf, Ta, W). The calculated CO_2_ and H adsorption energies (Figure 4e) reveal that Cr and Mo carbides exhibit strong hydrogen competition at ^*^C_lat_ sites, making them unsuitable for CO_2_ reduction. For the remaining systems, the critical selectivity‐determining step involves hydrogenation of the ^*^C_lat_HCH_2_ intermediate. While Hf, Ta, and W carbides show thermodynamic preference for forming ^*^C_2_H_4_ (Figure 4f), all systems exhibit prohibitively higher desorption energy for C_2_H_4_ (>0.88 eV) (Figure 4g) than the CH_4_ formation energy.

The kinetic limitations are further elucidated by examining the ^*^C_lat_HCH_2_ hydrogenation barrier (Figure 4h). The higher energy required for ^*^C_lat_HCH_2_→^*^C_2_H_4_ than that for ^*^C_lat_HCH_2_→^*^C_lat_HCH_3_ stems from fundamental electronic structure differences. The analysis of ^*^C_lat_HCH_2_ reveals that ^*^C_lat_ adopts sp^2^ hybridization (evidenced by Ti─C─Ti = 97.94° and Ti─C─H≈120° angles), while the adsorbed carbon maintains sp^3^ character (H─C─H = 108.5°) (Figure S10a). The sp^2^ hybridization state of ^*^C_lat_ and its corresponding orbital‐bonding relationships are depicted schematically in Figure S10b. This hybridization mismatch creates an intrinsic kinetic barrier—hydrogenating ^*^C_lat_ requires disrupting its stable sp^2^ configuration, whereas the adsorbed carbon's sp^3^ state readily accommodates additional hydrogen. The structural challenge is compounded by the requirement to break three metal‐carbon bonds for C_2_H_4_ desorption (Figure S10a), making this step prohibitively difficult even when thermodynamics favor ^*^C_2_H_4_ formation.

These limitations persist in V─Cr solid solution MXenes (Figure S11a–e), where improved ^*^C_2_H_4_ selectivity and reduced desorption energy remain offset by high hydrogenation barriers. Furthermore, the introduction of K⁺ ion into the solvent environment is also unable to lower the energy barrier for C_2_H_4_ formation (Figure S12). The combined thermodynamic and kinetic constraints explain why CO_2_ reduction at MXene ZZ1 overwhelmingly favors C_1_ products, despite the initial facile adsorption of CO_2_ at ^*^C_lat_ sites. The structural and electronic properties of the lattice carbon create an inherent selectivity toward CH_4_ formation that is difficult to overcome through simple metal substitution. This selectivity is robust and cannot be reversed even by substituting the common ‐OH termination with ‐F groups, which also fails to enable C_2_H_4_ production (Figure S13). DFT+U calculations on the ZZ1 of Ti_2_C(OH)_2_ show that while the adsorption energies of both CO_2_ and H are reduced, the selectivity between them remains unchanged (Figure S30a). More importantly, the key limitation persists: the lattice carbon atom cannot be extracted to form C_2_H_4_, even with the inclusion of the Hubbard U correction (Figure S30b,c).

In summary, while edge carbon atoms may interact with intermediates, they cannot be released from the MXene lattice to serve as a carbon source for C_2_H_4_ production in the CO_2_ reduction reaction based on our calculations.

### 3.3 C_2_ Generation Through CO Dimerization on ZZ3^*^ Edge

**FIGURE 5:**
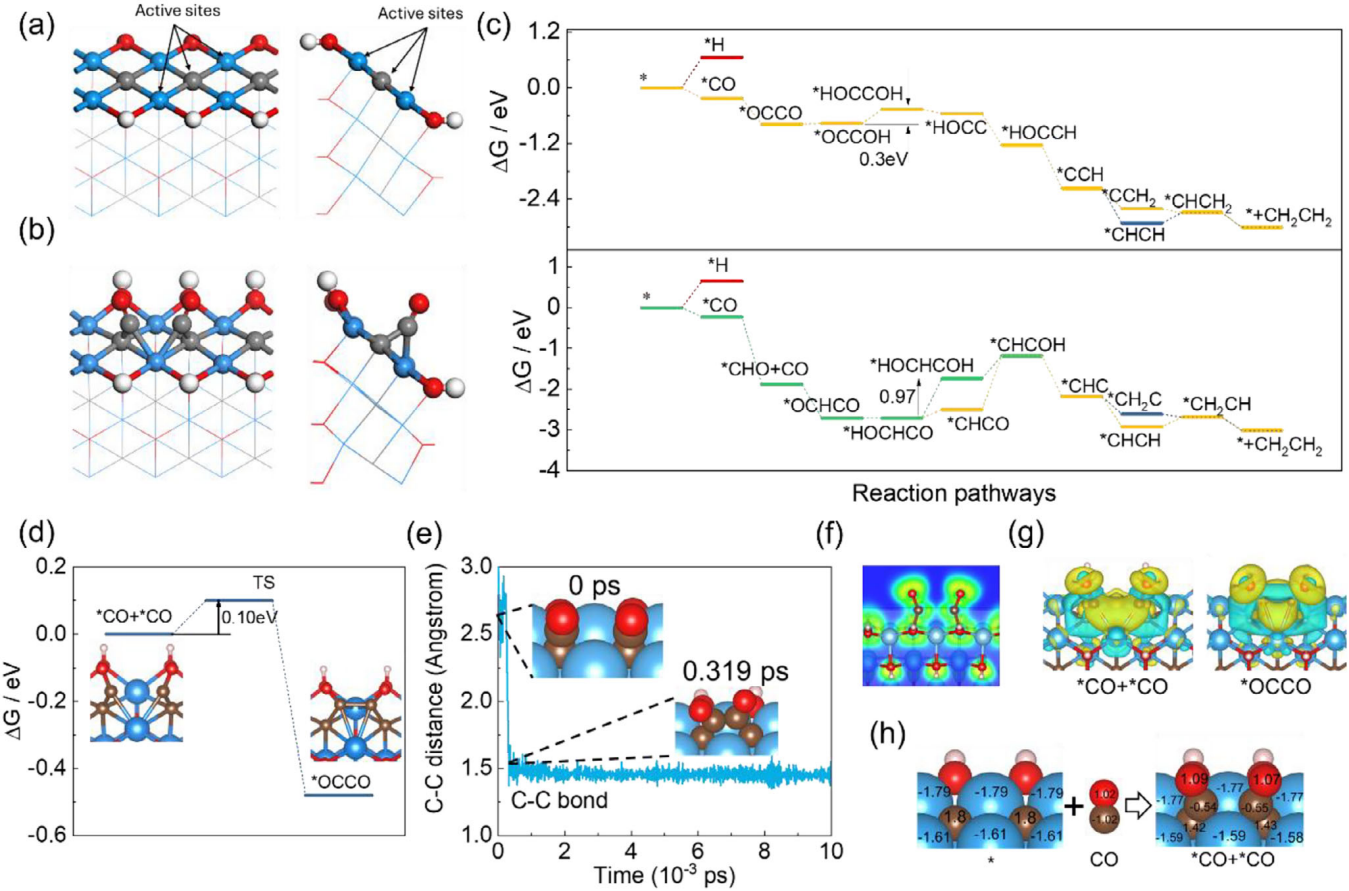
Structural and mechanistic analyses of CO reduction on Ti_2_C(OH)_2_‐ZZ3^*^. (a) Structure of ZZ3^*^. (b) Adsorption configuration of two adjacent CO. (c) Free energy diagram for CO reduction to C_2_H_4_ at 0 V vs. RHE. The upper and lower panels show the pathways via direct CO─CO coupling and via ^*^CHO intermediate, respectively. (d) C─C coupling barrier determined by climbing‐image nudged elastic band (CI‐NEB) calculations. (e) Evolution of C─C bond distance during explicit‐solvent AIMD simulation. (f) ELF analysis of adjacent ^*^CO. (g) Differential charge density in the ^*^CO‐dimerization process (isosurface = ±0.003 eÅ^−^^3^). (h) Bader charge evolution during the CO adsorption (positive/negative value indicates electron gain/loss).

Unlike ZZ1, ZZ3^*^ exhibits a distinct structural configuration with rich unsaturated Ti sites and increased Ti coordination around the ^*^C_lat_ atoms (Figure 5a). When CO_2_ adsorbs on this edge, it adopts a geometry in which the oxygen atoms bind to Ti sites while the carbon atom forms a covalent bond with ^*^C_lat_ (Figure S14a). However, the strong oxygen adsorption at Ti sites creates a substantial free energy barrier (0.95 eV) for the initial hydrogenation step in the CO_2_ reduction (Figure S14b). The thermodynamic analysis favors formic acid production over CO. More critically, the high CO_2_ activation energy (>1 eV) may lead to a prohibitive kinetic challenge (Figure S14c). Consequently, CO_2_ reduction is intrinsically difficult to proceed directly on the ZZ3^*^ edge.

Remarkably, ZZ3^*^ demonstrates exceptional activity for C─C coupling between adsorbed CO molecules, exhibiting a favorable free energy change of −0.56 eV (Figure 5c) and an extremely low kinetic barrier of 0.10 eV as determined by CI‐NEB calculations (Figure 5d). To confirm the reliability of this exceptionally low barrier, a series of rigorous validation calculations was performed. The supercell size was expanded to avoid spurious interactions between periodic images (Figures S15 and S16a). Subsequently, k‐point convergence tests were conducted, confirming that the barrier is well‐converged (Figure S16b). Furthermore, the vibrational frequencies of the transition state were calculated (Table S2). The presence of a single imaginary frequency, corresponding to the vibration along the C─C bond formation direction (Figure S17), unequivocally validates the identified transition state structure. DFT+U calculations yield a slightly higher C─C coupling barrier (0.4 eV compared to 0.1 eV without U, Figure S30d) and a more negative reaction free energy. Despite these quantitative shifts, the fundamental conclusion, that is, C─C coupling on this edge is both thermodynamically favorable and kinetically facile, remains robust and unchanged.

Moreover, CO exhibits stronger competitive adsorption than H on this edge, which further favors the coverage of reactive CO intermediates. The facile coupling is further supported by AIMD simulations in explicit solvent, where the formation of C─C bond occurs within 0.3 ps and remains stable throughout the simulation (Figure 5e). This efficient coupling is also maintained in the presence of K⁺ ions, as confirmed by additional simulations in an explicit cationic environment (Figure S18). The subsequent hydrogenation steps proceed with a relatively small barrier, predominantly through spontaneous reactions except for the oxygen hydrogenation step (ΔG_(*OCCOH‐*HOCCOH)_ = 0.3 eV, Figure 5c, upper panel), ultimately leading to C_2_H_4_ production. In addition, we calculated an alternative pathway, where one ^*^CO is firstly hydrogenated to ^*^CHO before coupling. Although C─C coupling becomes even more favorable after hydrogenation, the subsequent hydrogenation of the resulting ^*^HOCHCO intermediate faces a prohibitively high barrier of 0.97 eV, rendering this pathway non‐competitive (Figure 5c, lower panel). Therefore, we conclude that CO prefers to dimerize prior to hydrogenation.

The ELF analysis of adsorbed ^*^CO–^*^CO pairs (Figure 5f) shows pre‐coupling electron localization between adjacent C atoms, while the differential charge density (Figure 5g) demonstrates charge accumulation between CO carbon atoms and corresponding depletion at the Ti_2_C(OH)_2_ edge. Quantitative Bader charge analysis (Figure 5h) confirms substantial electron transfer, with the CO carbon gaining 0.48 electrons and the ^*^C_1at_ atom losing 0.38 electrons upon adsorption. These findings collectively demonstrate that ZZ3^*^ provides an electron‐rich environment that enables efficient C─C coupling through significant charge transfer from the MXene edge to adsorbed CO species, making it highly effective for promoting multi‐carbon product formation despite the limitations for direct CO_2_ activation.

Subsequently, we calculated the CO coupling barriers on the ZZ3^*^ edges of Ti_2_CO_2_, yielding values of ∼0.4 eV (Figure S19a). In contrast, no C─C coupling was observed on the ZZ3 edge of Ti_2_CF_2_ within the convergence criteria of our calculations (Figure S19c). This distinct behavior may stem from the strong electronegativity of the F terminal groups, which occupy Ti sites and significantly reduce the electron density available on the adjacent lattice carbon atoms, thereby impeding the charge transfer required for the coupling. We further examined the effect of MXene thickness on the coupling barrier. As the number of metal layers increases, the barrier rises to approximately 0.6–0.75 eV (Figure S20). In contrast, the C─C coupling on the ZZ1 of Ti_2_C(OH)_2_ is significantly hindered, with an endergonic free energy change of 1.57 eV (Figure S21a). Differential charge density analysis reveals a less pronounced charge accumulation at the ZZ1 compared to ZZ3^*^ (Figure S21b). Bader charge analysis shows that the lattice carbon at ZZ1 carries a charge of 0.64 |e| after CO adsorption, markedly lower than the value of 1.42 |e| observed at the ZZ3^*^ edge (Figure S21c). This substantial difference in the electronic environment likely accounts for the difficulty of CO coupling at the ZZ1.

**FIGURE 6:**
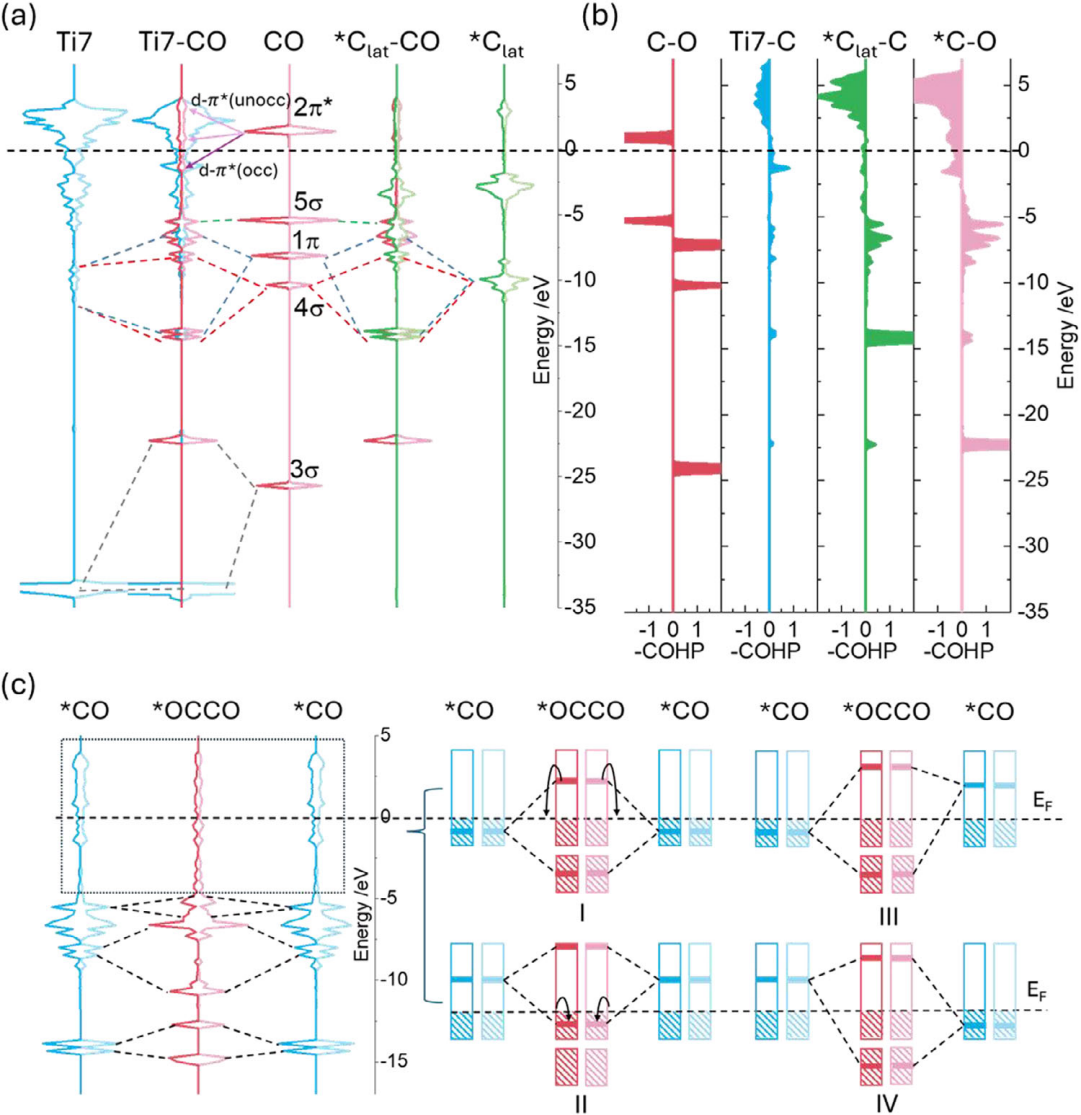
Electronic structure analysis. (a) PDOSs of Ti7 (adsorption metal site), ^*^C_lat_ (lattice carbon site), and CO gas molecule, and their interaction within ZZ3^*^‐CO. (b) pCOHP analysis for C─O (gas molecule), Ti─C (CO), ^*^C_lat_‐C (CO), and ^*^C─O (adsorbed CO). (c) PDOSs of C atoms in adsorbed CO (^*^CO) and coupled ^*^OCCO, and the schematic illustration of the interaction between the 2π^*^ orbitals of ^*^CO.

To elucidate how electron‐rich lattice carbon promotes C─C coupling, we analyzed the electronic structure of CO adsorbed on MXene edge (Figure 6a). CO bridges Ti and ^*^C_lat_ (Figure 5b), where its 3σ orbital couples with Ti 3p orbitals and shifts to higher energy. The 4*σ* and 1*π* orbitals predominantly hybridize with the s orbitals of ^*^C_lat_, strengthening ^*^C_lat_─CO bonds, while the 5*σ* orbital remains unperturbed. The 2*π* ^*^ orbital interacts with Ti 3d orbitals, splitting into occupied and unoccupied d–*π* ^*^ states. The COHP analysis further corroborates the pDOS results (Figure 6b). It reveals that the bonding orbitals of the adsorbed CO are primarily located near the Fermi level for its interaction with Ti atoms, but are centered between −15 and −5 eV for its interaction with the ^*^C_lat_ site. Furthermore, the partial filling of the corresponding antibonding orbitals in the adsorbed CO molecule confirms our initial hypothesis regarding antibonding‐state population. The 2*π* ^*^ orbital filling weakens the C≡O bond (ICOHP in Figure S22a; elongated bond in Figure S22b) and enables C─C coupling.

Near E_F_, diffused 2*π* ^*^ orbitals facilitate C─C coupling through three interaction pathways (Figure 6c): (I) occupied‐occupied orbital mixing forms a bonding state (stabilized by depopulating its antibonding partner above E_F_); (II) unoccupied‐unoccupied interactions generate low‐energy acceptor orbitals that become populated; (III/IV) occupied‐unoccupied hybridization directly yields bonding states. Frontier orbital theory demonstrates that 2π^*^ orbitals proximal to the E_F_ exert the dominant influence on bonding. Bader charge analysis (Figure 5h) indicates that electron transfer from Ti to lattice carbon is diverted to CO, depleting ^*^C_lat_ charge while minimally altering Ti charge.

**FIGURE 7:**
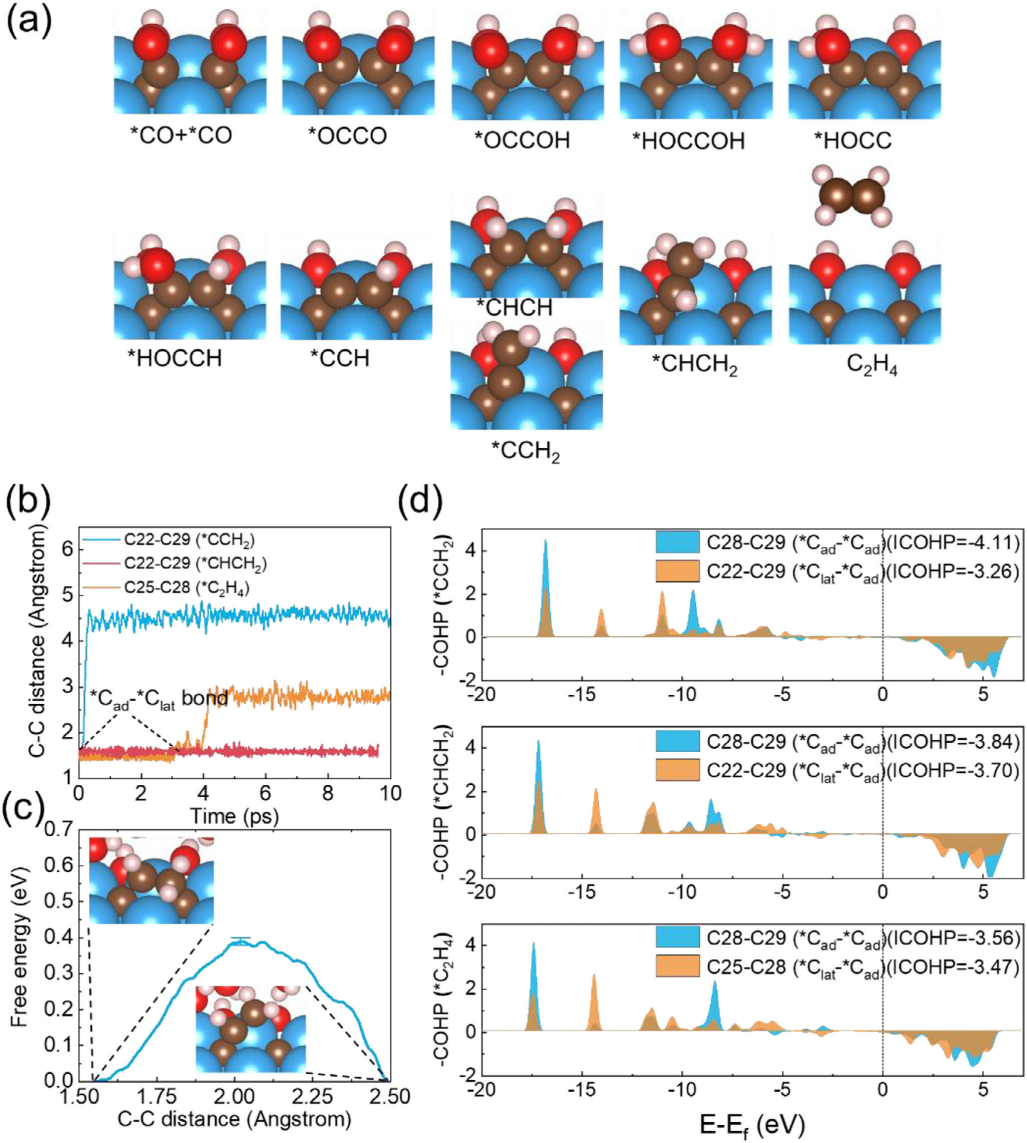
C─C bond stability in CO reduction on Ti_2_C(OH)_2_ ZZ3^*^. (a) Intermediates along the CO reduction pathway. (b) AIMD evolution of ^*^C_ad_─^*^C_lat_ bond distances (^*^CCH_2_, ^*^CHCH_2_, ^*^C_2_H_4_). (c) Cleavage energy profile of ^*^CHCH_2_ via constrained MD. (d) pCOHP analysis: bonding interactions in (i) ^*^C_ad_─^*^C_ad_ vs. (ii) ^*^C_ad_─^*^C_lat_ for all intermediates.

To ensure the viability of C_2_ product formation, we systematically evaluated the desorption capability of C_2_H_4_ by analyzing the relative bond strengths between adsorbed carbon (^*^C_ad_) pairs (^*^C_ad_═^*^C_ad_) vs. adsorbed‐lattice carbon pairs (^*^C_ad_═^*^C_lat_). The critical intermediates involved in breaking the ^*^C_ad_═^*^C_lat_ bond (^*^CCH_2_, ^*^CHCH_2_, and ^*^C_2_H_4_) were identified through structural analysis (Figure 7a). Explicit‐solvent AIMD simulations reveal distinct bond dissociation behaviors: the ^*^C_ad_═^*^C_lat_ bond in ^*^CCH_2_ (C22═C29) breaks immediately (blue curve, Figure 7b), while ^*^C_2_H_4_ (C25–C28) is dissociated after ∼4 ps (orange curve). Notably, the ^*^CHCH_2_ intermediate (C22═C29) maintains its ^*^C_ad_═^*^C_lat_ bond throughout the 10 ps simulation (red curve), establishing the bond strength hierarchy: ^*^CHCH_2_> ^*^C_2_H_4_> ^*^CCH_2_.

The barrier for breaking the ^*^C_ad_═^*^C_lat_ bond in ^*^CHCH_2_ is calculated to be ∼0.4 eV by the constrained molecular dynamics simulations (Figure 7c), confirming facile dissociation under reaction conditions. Complementary crystal orbital Hamilton population (COHP) analysis (Figure 7d) shows that the more negative integrated COHP (ICOHP) values for ^*^C_ad_═^*^C_ad_ bonds vs. ^*^C_ad_═^*^C_lat_ bonds across all intermediates give stronger bonding between adsorbed carbons.

The trend for the ICOHP‐derived bond strength (^*^CHCH_2_> ^*^C_2_H_4_> ^*^CCH_2_) perfectly matches the AIMD observations. Crucially, the combined results demonstrate that the ^*^C_ad_═^*^C_ad_ bond in ^*^C_2_H_4_ becomes significantly stronger than the ^*^C_ad_═^*^C_lat_ bond during the reduction, enabling efficient product desorption—a critical requirement for sustained catalytic turnover.

We systematically evaluated CO_2_ reduction at ZZ3^*^ sites across metal‐based MXenes. Adsorption free energies show CO_2_ generally dominates over H at these edges, except for Mo‐/W‐carbides where high metal electronegativity favors H occupation of C sites (Figure S23a). Strong CO_2_ adsorption at most metal sites imposes >1 eV barriers for CO_2_→COOH hydrogenation, particularly in Ta‐, Hf‐, Nb‐, Ti‐, and V‐based systems (Figures S23b and S24a–e). Although Zr‐/Cr‐carbides exhibit lower hydrogenation barriers, their C_2_ selectivity is poor (Figure S25a,b): Cr_2_C(OH)_2_ favors HCOOH formation, while Zr_2_C(OH)_2_ shows prohibitively high CO coupling barriers.

**FIGURE 8:**
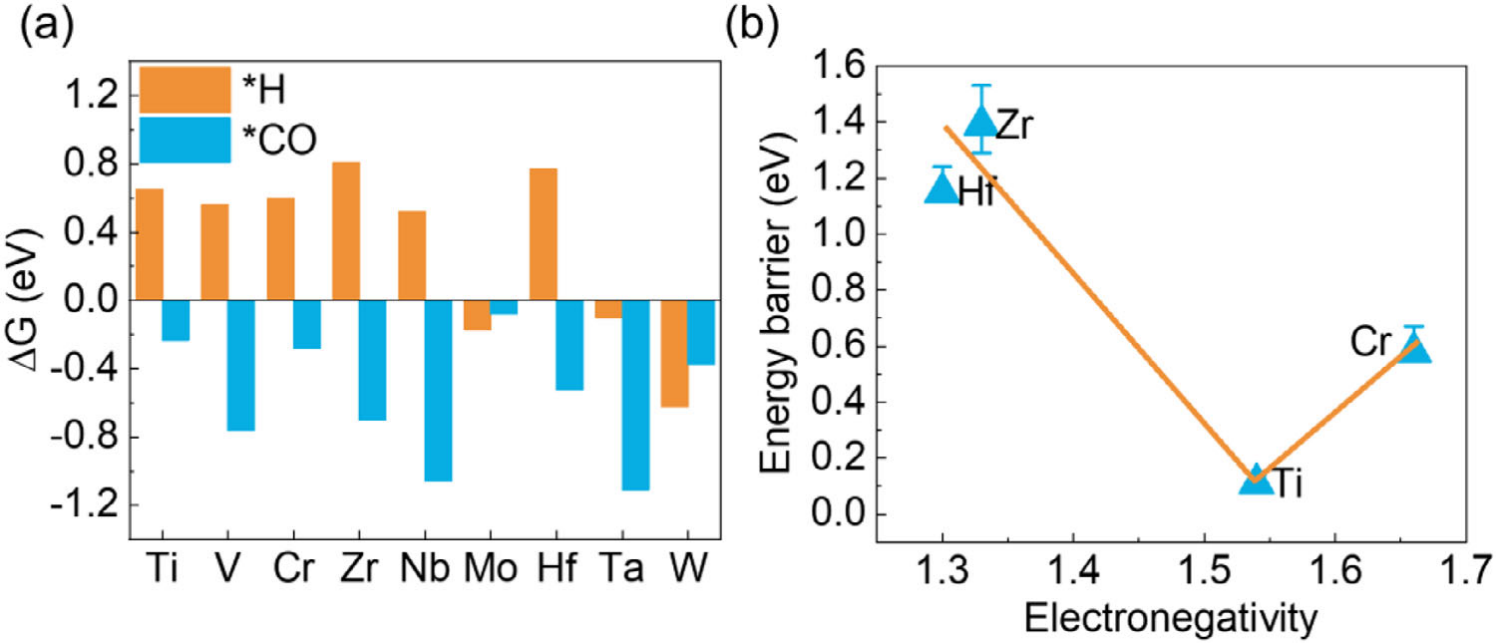
Comparative energetics of adsorption and reaction on various MXenes. (a) H vs. CO competitive adsorption. (b) Inverse volcano relationship between C─C coupling barriers and transition metal electronegativity, with Ti_2_C(OH)_2_ at the optimal minimum.

Shifting focus to CO reduction performance, we first examined the competitive adsorption between CO and H (Figure 8a). Mo and W carbides again show preferential H adsorption, while V‐, Nb‐, and Ta‐ carbides strongly adsorb CO at metal sites (Figure S26a–c). This metal‐site adsorption suppresses electron population in the CO 2*π* ^*^ orbital (Figure S27). Upon adsorption, the CO 2*π* ^*^ orbital shifts to higher energies without broadening, retaining high localization and predominantly unoccupied character above E_F_. Such electronic configuration is intrinsically unfavorable for C─C coupling.

For Cr, Zr, and Hf carbides, detailed CO coupling analyses reveal intriguing trends (Figure 8b; Figure S28a). Despite the lattice carbon atoms getting more electrons from coordination metal atoms (as evidenced by Bader charge analysis, Figure S28b), Hf and Zr carbides exhibit higher CO coupling barriers than Ti_2_C(OH)_2_. This counterintuitive result stems from their metals’ lower electronegativity, which causes strong dual adsorption (both C and O atoms) that sterically hinders coupling (Figure S28c). Conversely, Cr's higher electronegativity results in insufficient electron surrounding the lattice carbon, also leading to elevated coupling barriers. These comprehensive comparisons demonstrate that Ti_2_C(OH)_2′_s ZZ3^*^ achieves an optimal balance of electronic and steric factors, exhibiting the most favorable performance for electrocatalytic CO reduction to C_2_H_4_ among all studied MXenes.

## 4 Conclusion

In summary, our comprehensive study elucidates the pivotal role of lattice carbon in CORR catalysis at bilayer MXene edges. While lattice carbon cannot be released as a carbon source in CO_2_ reduction to form C_2_ products, it mediates a novel CO coupling mechanism that drives efficient electrocatalytic CO reduction to C_2_H_4_. The ZZ3^*^ edge of Ti_2_C(OH)_2_ exhibits exceptional performance, with electron donation to adsorbed CO species via electron‐rich lattice carbon sites, resulting in an ultralow C─C coupling barrier of 0.10 eV. By populating CO 2*π* ^*^‐orbitals, electrons weaken C≡O bonding and lower the kinetic barrier for C─C coupling. This mechanism is supported by favorable bond strength dynamics, where stronger carbon–carbon bonds facilitate efficient product desorption compared to weaker carbon‐lattice interactions. Comparative analyses across MXene systems highlight metal electronegativity as a critical determinant of charge distribution at lattice carbon sites, with Ti_2_C(OH)_2_ positioned at the peak of an inverse volcano relationship for CO coupling barriers due to its balanced electronic properties. These insights establish lattice carbon‐mediated coupling as a distinct and effective pathway, diverging from conventional metal‐centric mechanisms. By providing both fundamental understanding of the role of carbon in electrocatalysis and actionable design principles for high‐performance C_2_ production catalysts, this work paves the way for the rational engineering of MXene edge structures and electronic environments, offering a promising strategy for sustainable fuel and chemical synthesis.

## Conflicts of Interest

The authors declare no conflicts of interest.

## Supporting information




**Supporting File**: advs73535‐sup‐0001‐SuppMat.docx.

## Data Availability

The data that support the findings of this study are available from the corresponding author upon reasonable request.
