# Supplementary material for: Lattice Carbon‐Mediated Ultralow‐Barrier C–C Coupling for Selective CO Electroreduction to Ethylene
Source: Adv Sci (Weinh). 2025 Dec 23;13(14):e21983. doi: 10.1002/advs.202521983 (PMC12970250; doi:10.1002/advs.202521983)
Supplement: Supplementary file 1 — Supporting File: advs73535‐sup‐0001‐SuppMat.docx. [file ADVS-13-e21983-s001.docx]

Supporting Information

**Lattice Carbon-Mediated Ultralow-Barrier C-C Coupling for Selective CO Electroreduction to Ethylene**

Jiangke Tao^1^, Zhichao Yu^1^, Lulu Chen^2, 3^, Weng Fai Ip^4*^, Sen Lin^2,3^ and Hui Pan^1,4*^

^1^Institute of Applied Physics and Materials Engineering, University of Macau, Macao SAR, 999708 P. R. China

^2^State Key Laboratory of Photocatalysis on Energy and Environment, College of Chemistry, Fuzhou University, Fuzhou, 350116 P. R. China

^3^State Key Laboratory of Chemistry for NBC Hazards Protection, College of Chemistry, Fuzhou University, Fuzhou, 350116 P. R. China

^4^Department of Physics and Chemistry, Faculty of Science and Technology, University of Macau, Macao, SAR, 999078 P. R. China

*To whom correspondence should be addressed: [andyip@um.edu.mo](mailto:andyip@um.edu.mo); [huipan@um.edu.mo](mailto:huipan@um.edu.mo)


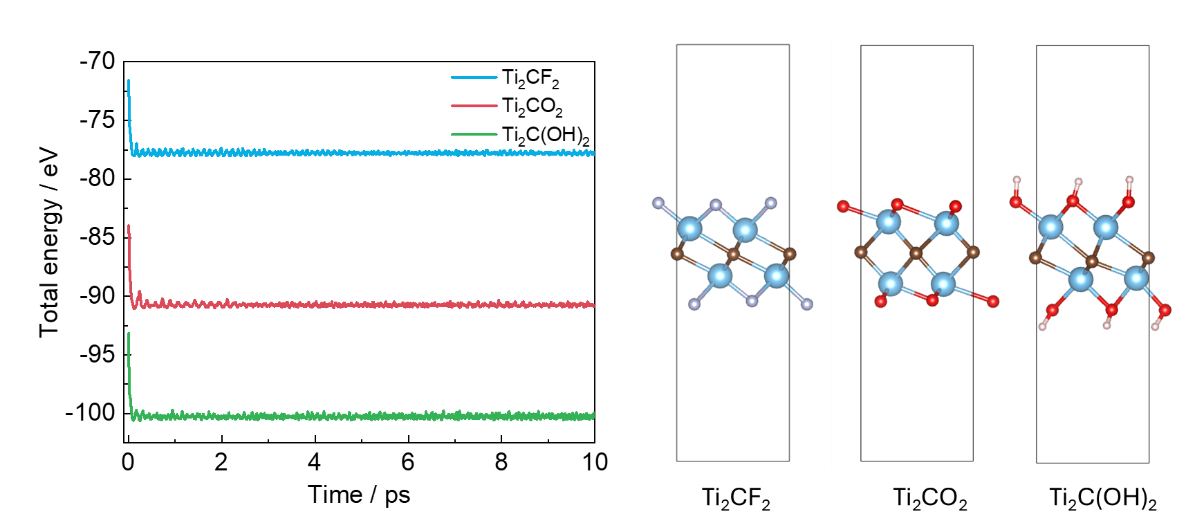


Fig. S1 Evolution of total energy of Ti_2_CF_2_, Ti_2_CO_2_ and Ti_2_C(OH)_2_ at 300K over time. Three MXenes with different functional groups remained stable in a vacuum environment.


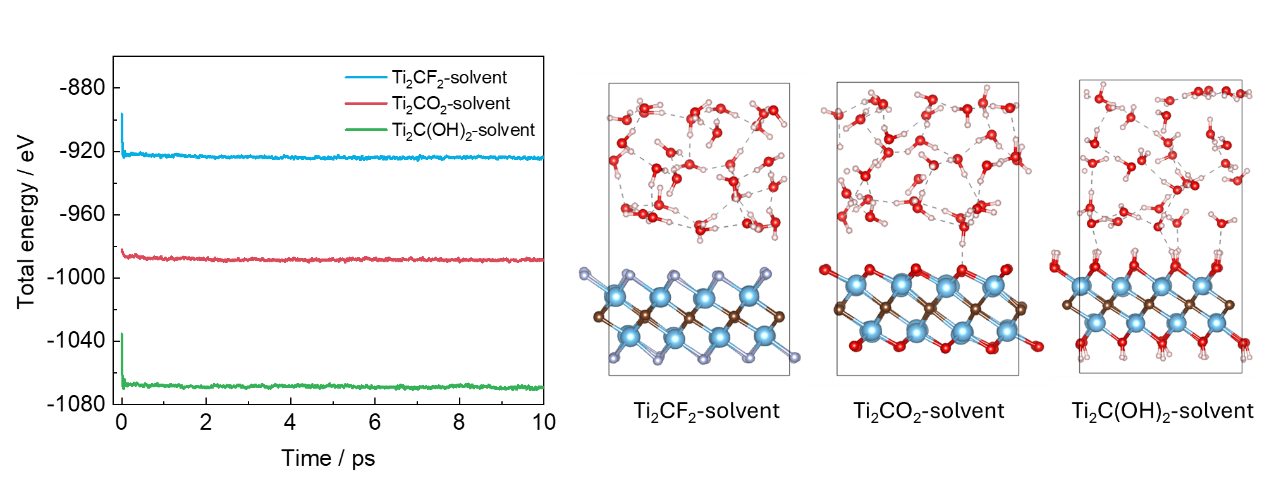


Fig. S2 Evolution of total energy of Ti_2_CF_2_, Ti_2_CO_2_ and Ti_2_C(OH)_2_ at 300K over time in solvent environment. Three MXenes with different functional groups remained stable in a solvent environment.


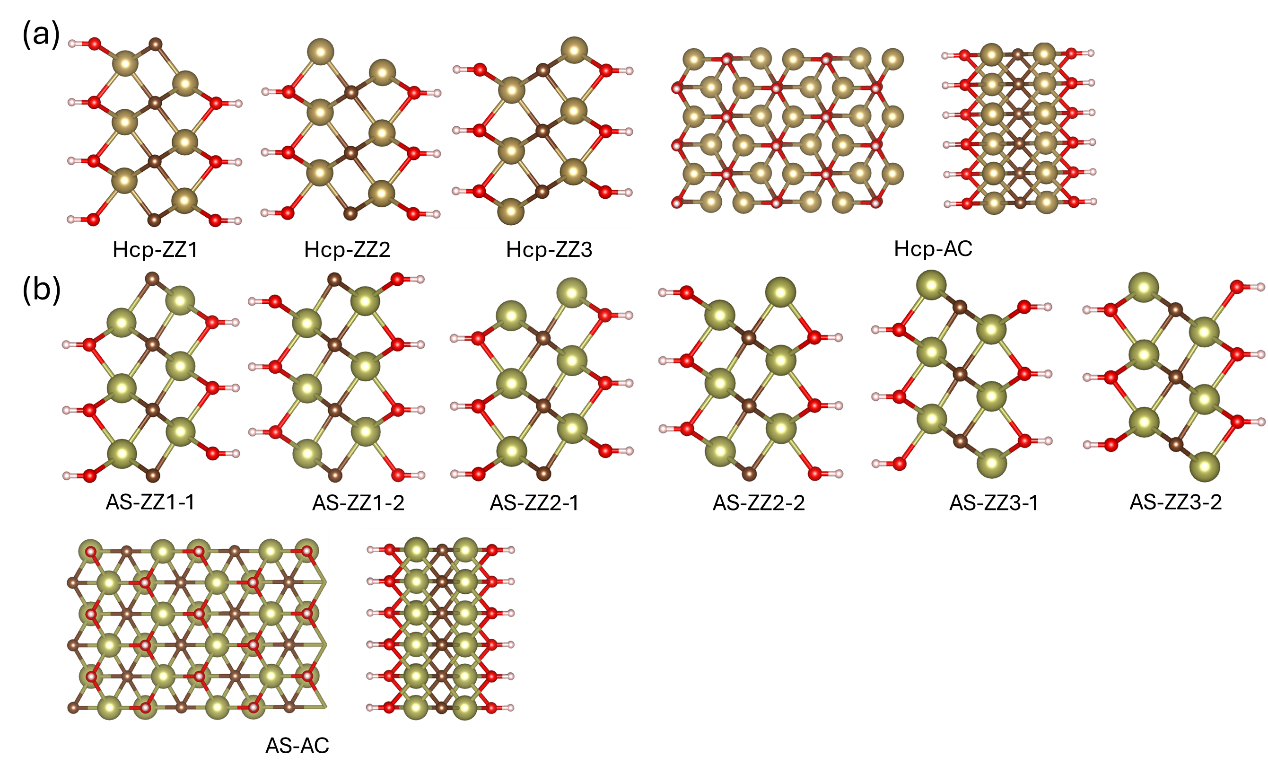


Fig. S3 (a) Four characteristic edges (ZZ1-ZZ3&AC) in hcp-type OH-functionalized Ta_2_C(OH)_2_. (b) Asymmetric OH-functionalized Hf_2_C(OH)_2_ edge structures. All edge structures are classified using the nomenclature established for fcc-type Ti_2_C(OH)_2_: ZZ1 denotes edges with exposed lattice carbon atoms, ZZ2 indicates buried carbon configurations, and ZZ3 represents coplanar carbon-metal arrangements.


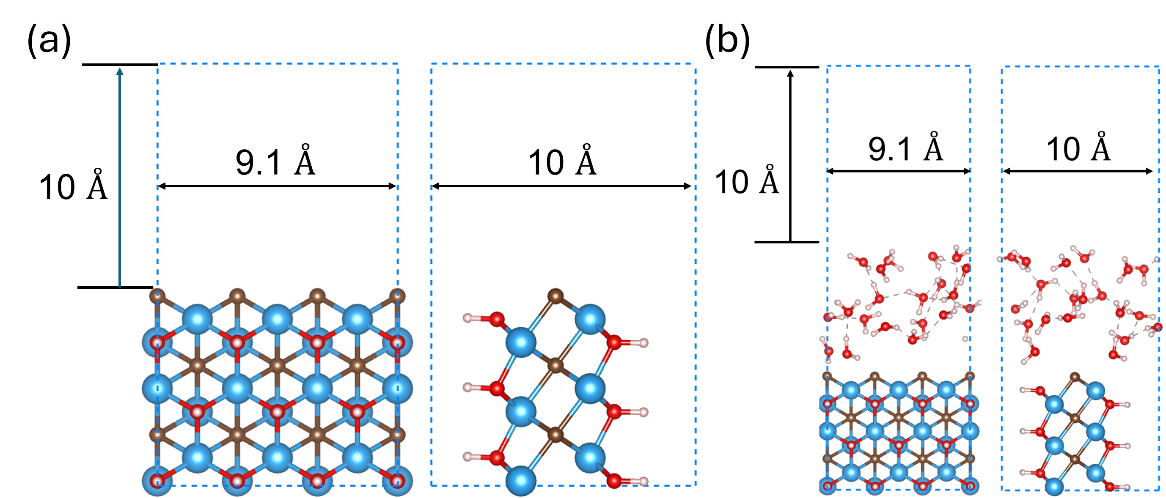


Fig. S4 (a) Supercell configuration for DFT calculations. (b) Explicit solvent model employed in AIMD simulations. The lower two layers of metal atoms are fixed.


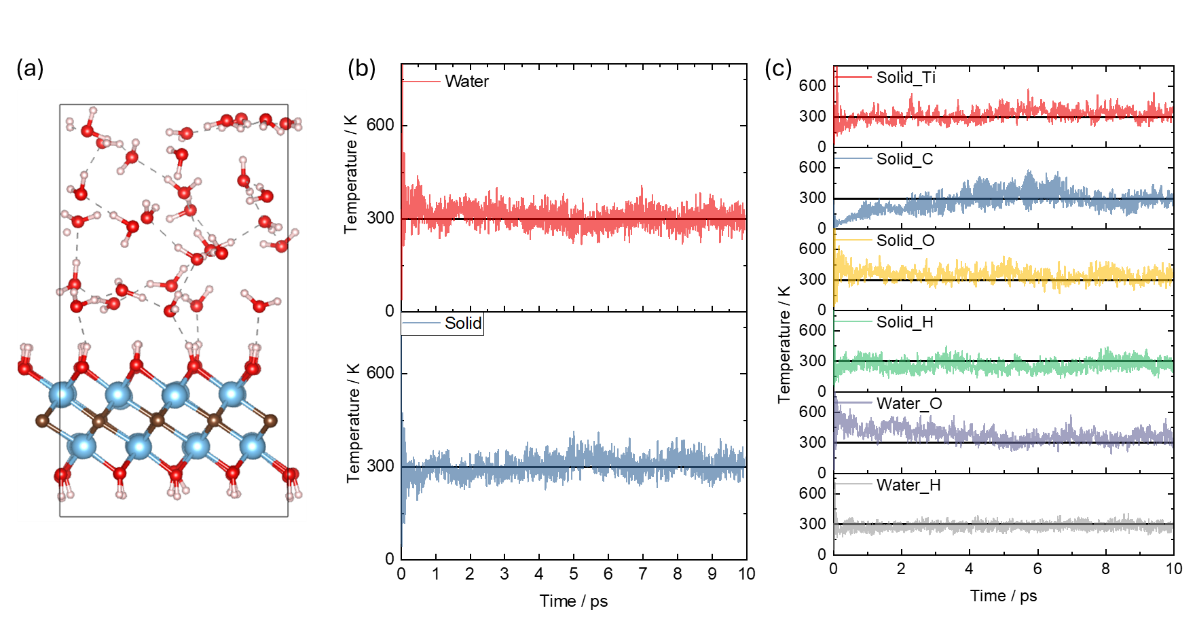


Fig. S5 (a) A snapshot of Ti_2_C(OH)_2_ in a solvent environment during simulation. (b) The temperature of the solvent molecules and the temperature of Ti_2_C(OH)_2_ during the simulation. (c) Temperature of different atoms (Ti, C, O and H) during the simulation. O and H atoms are divided into O and H atoms in MXene, and O and H atoms in water solvent.


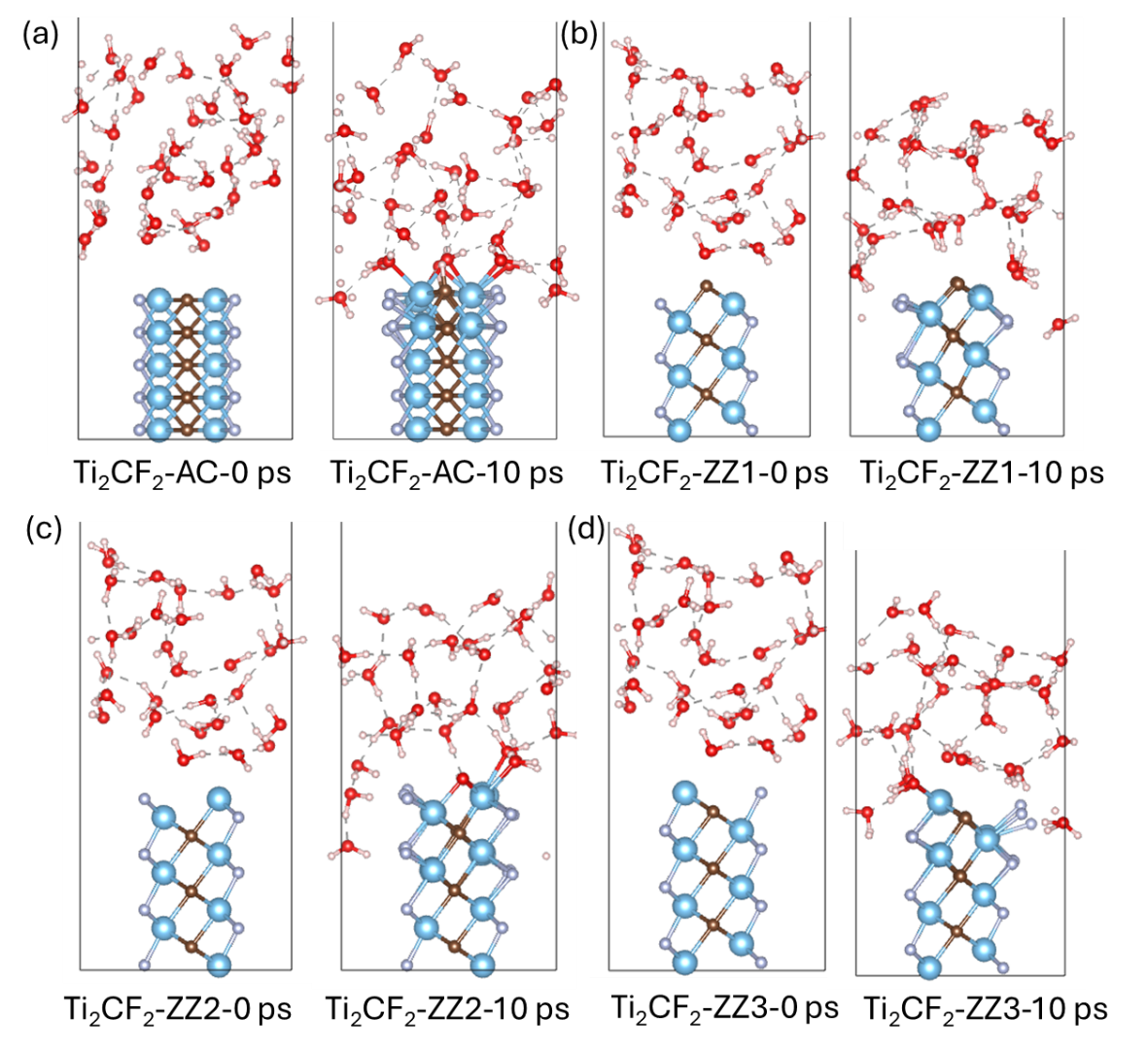


Fig. S6 Evolution of (a) AC, (b) ZZ1, (c) ZZ2 and (d) ZZ3 of Ti_2_CF_2_ at 300K in solvent environment over 10 ps.


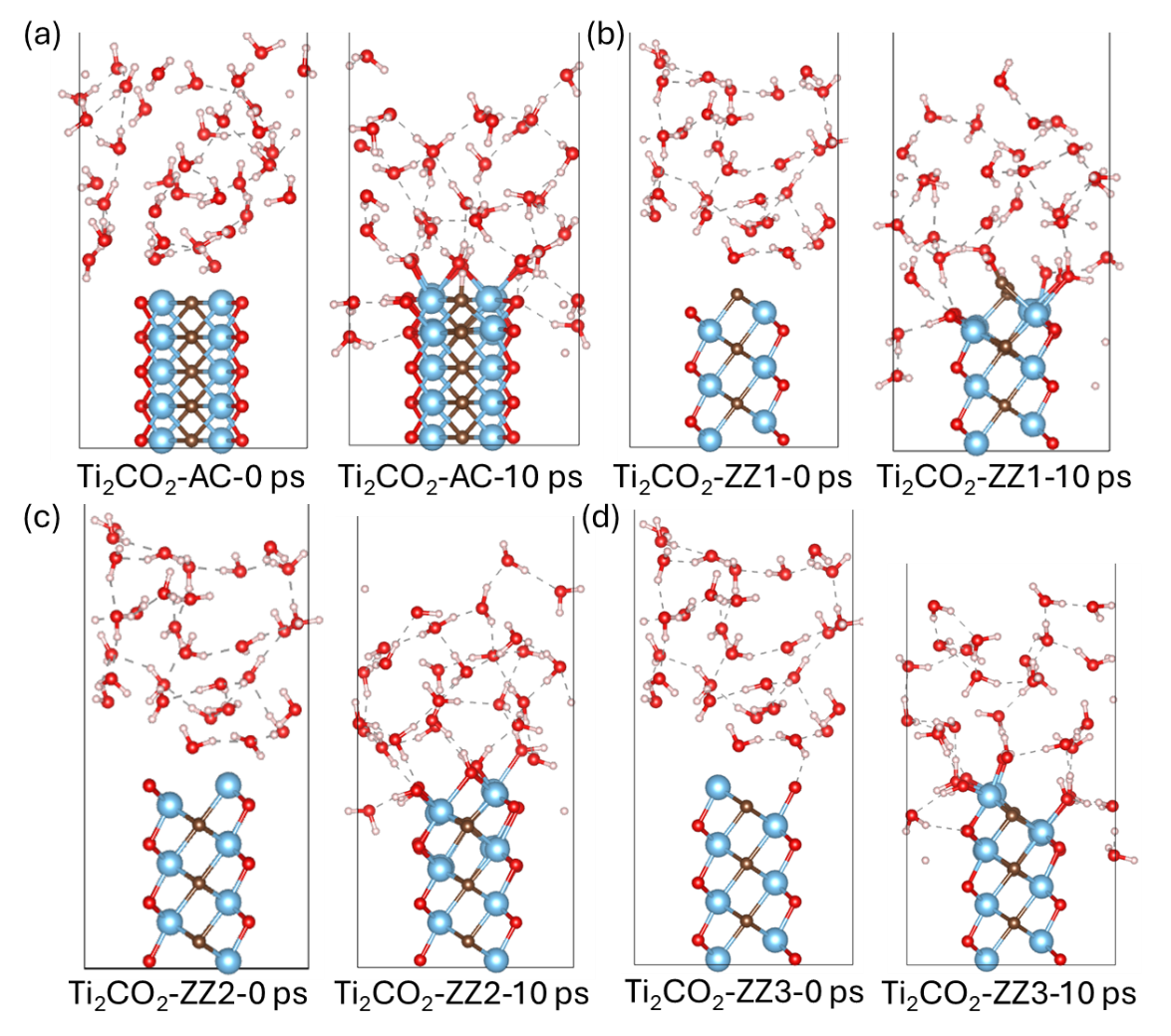


Fig. S7 Evolution of (a) AC, (b) ZZ1, (c) ZZ2 and (d) ZZ3 of Ti_2_CO_2_ at 300K in solvent environment over 10 ps.


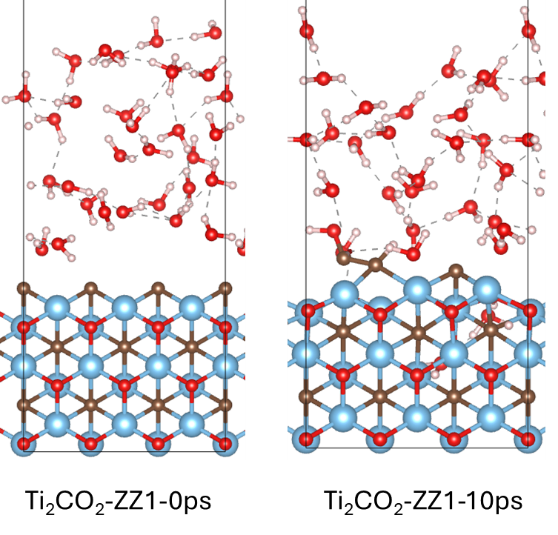


Fig. S8 Structural change of Ti_2_CO_2_ ZZ1 in solvent environment after 10 ps evolution.


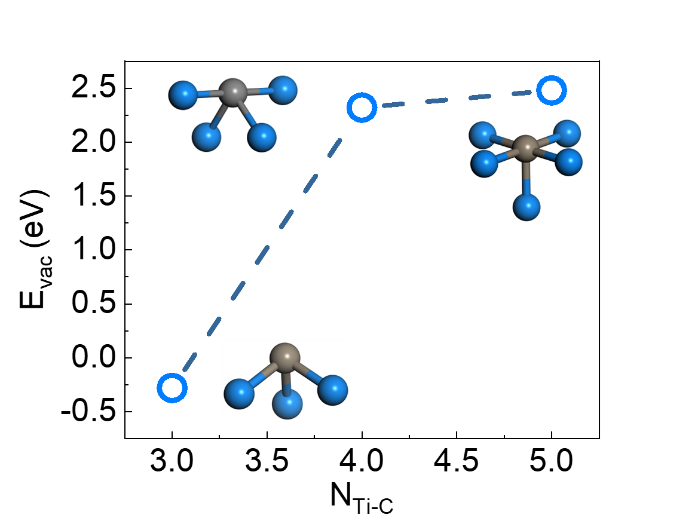


Fig. S9 The relationship between the C vacancy formation energy at the edge of Ti_2_C(OH)_2_ and the number of Ti-C bonds.


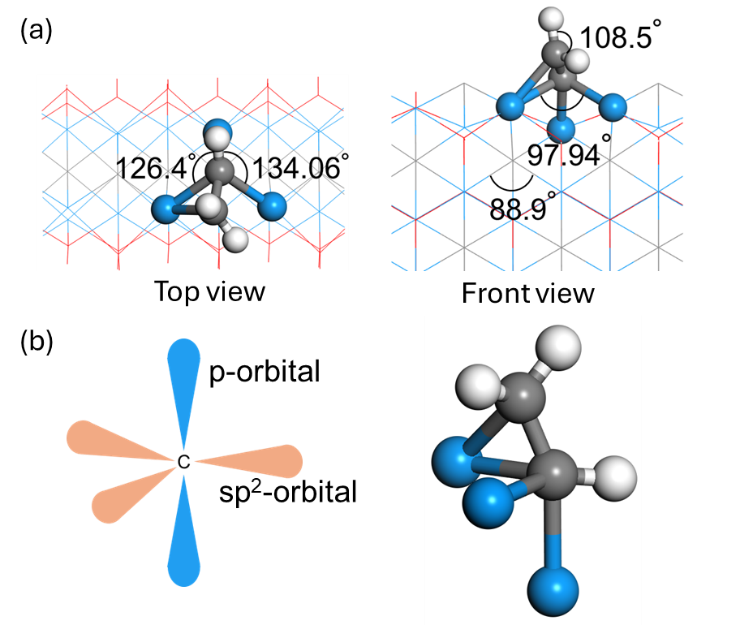


Fig. S10 (a) Optimized geometry of *C_lat_HCH_2_ intermediate showing key bond angles. (b) Schematic diagram of lattice carbon sp^2^ hybridization and the bonding atoms corresponding to each orbital.


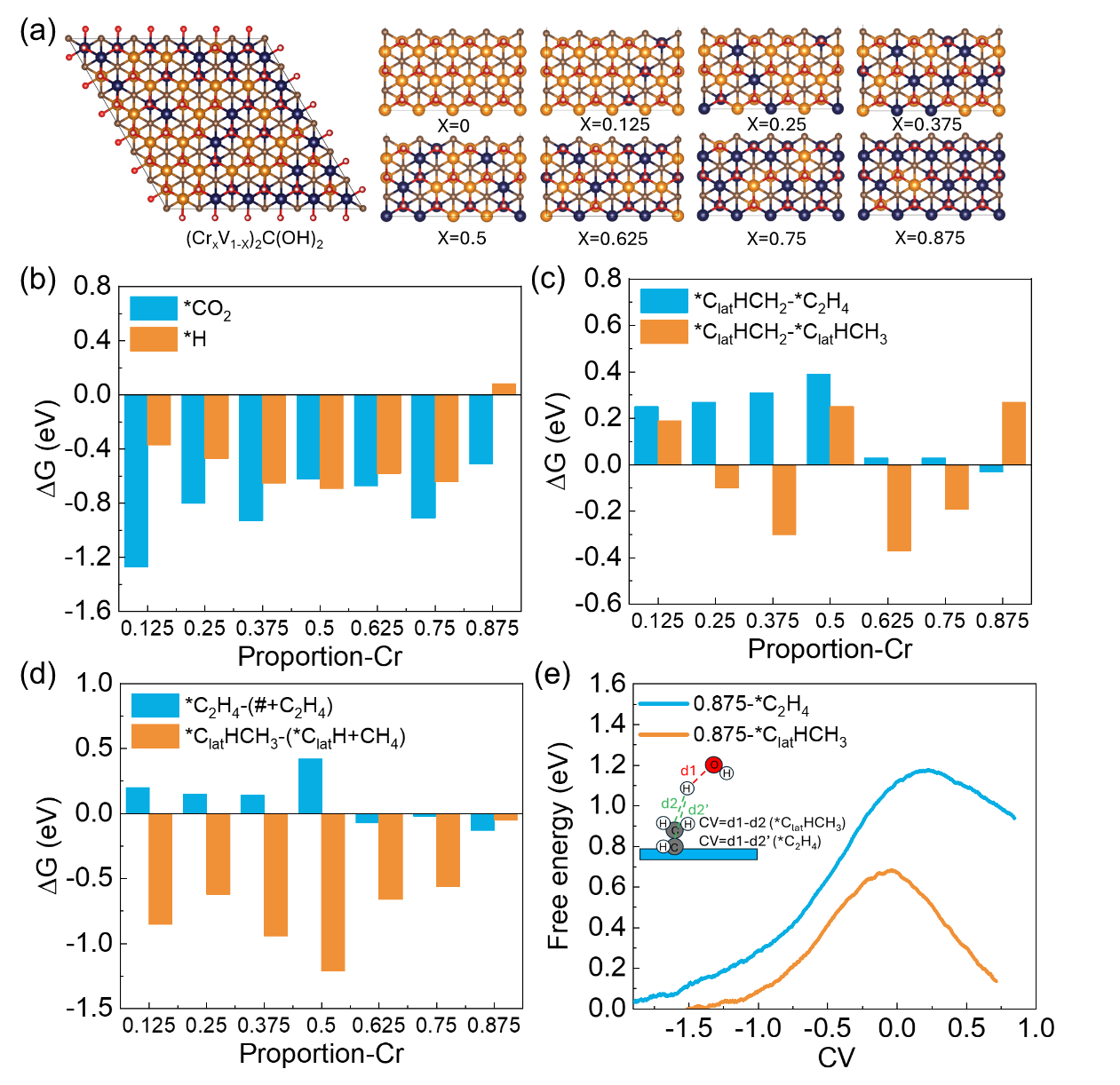


Fig. S11 Composition-dependent catalytic properties of (Cr_x_V_1-x_)_2_C(OH)_2_ solid solutions. (a) Disordered structure and representative ZZ1 configurations with varying Cr contents (x = 0-1). (b) Competitive adsorption free energies of *CO_2_ versus *H at ZZ1 across different Cr/V ratios. (c) Gibbs free energy changes for *C_lat_HCH_2_ hydrogenation pathways, demonstrating enhanced *C_2_H_4_ selectivity (C_2_ pathway) at x = 0.875. (d) Comparative free energy profiles for C_2_H_4_ desorption versus CH_4_ formation, showing reduced C_2_ product release difficulty at x = 0.875. (e) Kinetic barriers for competing *C_lat_HCH_2_ hydrogenation routes on (Cr_0.875_V_0.125_)_2_C(OH)_2_ ZZ1, with *C_2_H_4_ formation (blue) and *C_lat_HCH_3_ formation (orange).


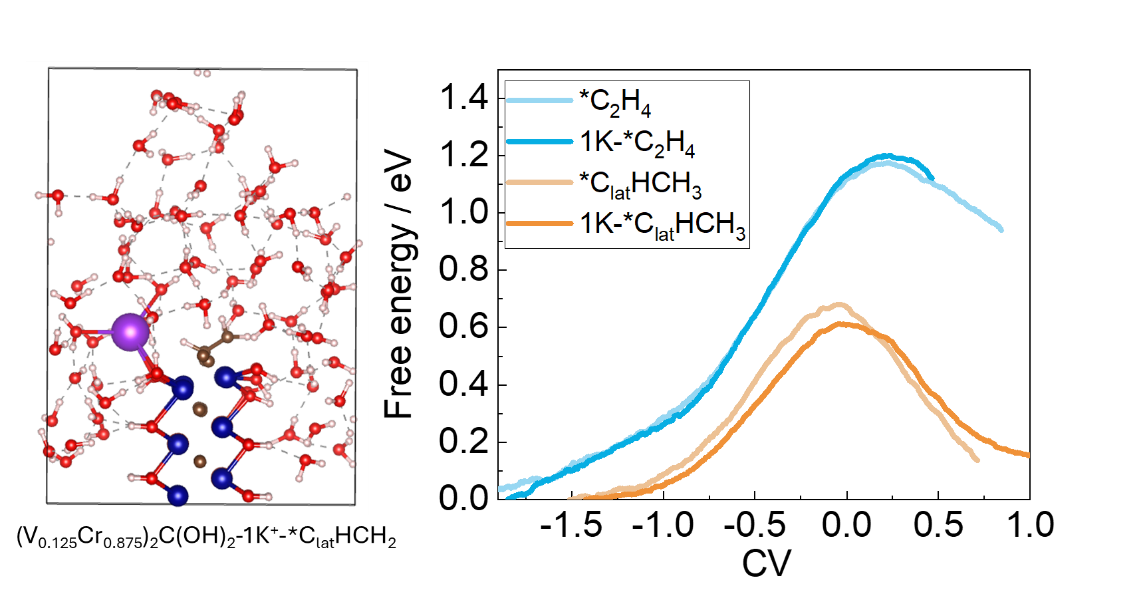


Fig. S12 Kinetic barriers for competing *C_lat_HCH_2_ hydrogenation routes on (Cr_0.875_V_0.125_)_2_C(OH)_2_ ZZ1 in solvent environment with and without one K^+^, with *C_2_H_4_ formation (blue for solvent environment with K^+^ and light blue for pure water solvent environment) and *C_lat_HCH_3_ formation (orange for solvent environment with K^+^ and light orange for pure water solvent environment).


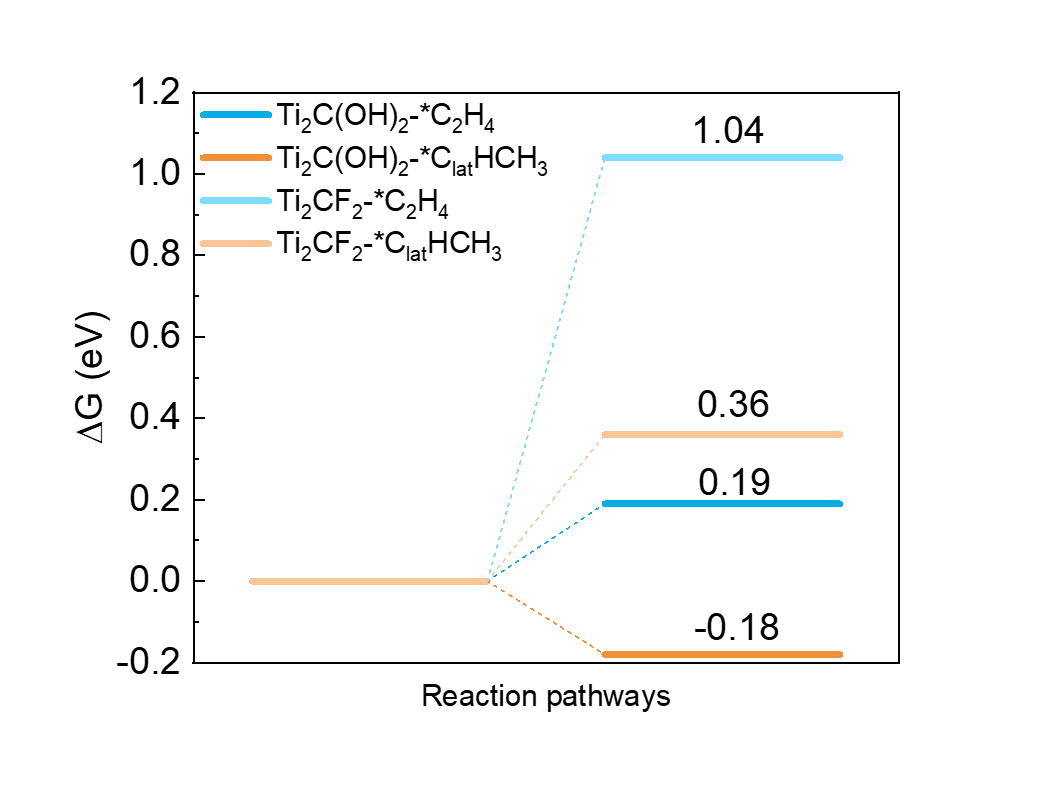


Fig. S13 Comparison of free energy changes from *C_lat_HCH_2_ to *C_lat_H_2_CH_2_ and *C_lat_HCH_3_ on the ZZ1 of Ti_2_CF_2_ and Ti_2_C(OH)_2_.


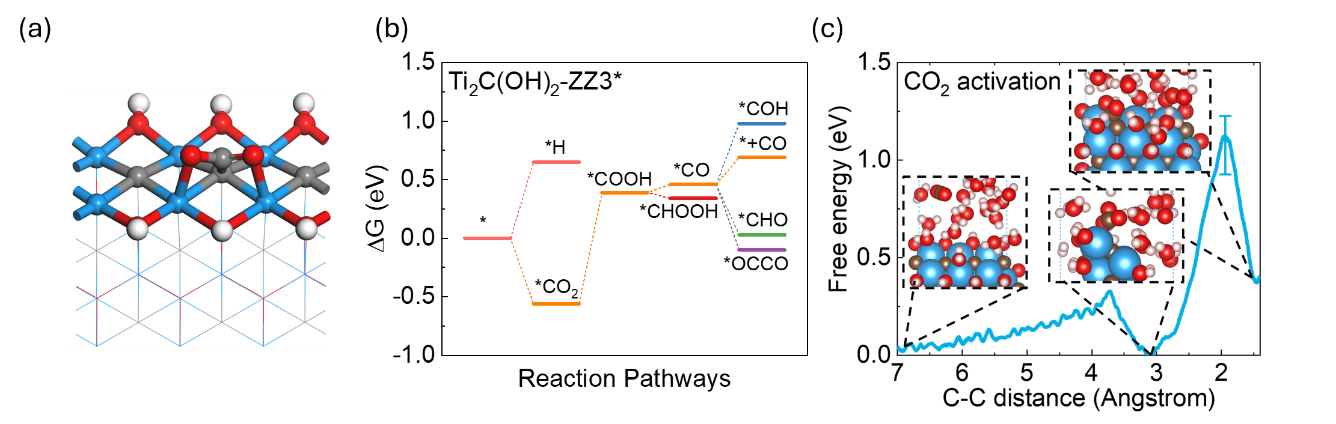


Fig. S14 (a) CO_2_ adsorption configuration. (b) Free energy diagram of CO_2_ reduction pathways. (c) Activation energy profile for CO_2_ adsorption. The insets show the configuration of CO_2_ during the activation process.


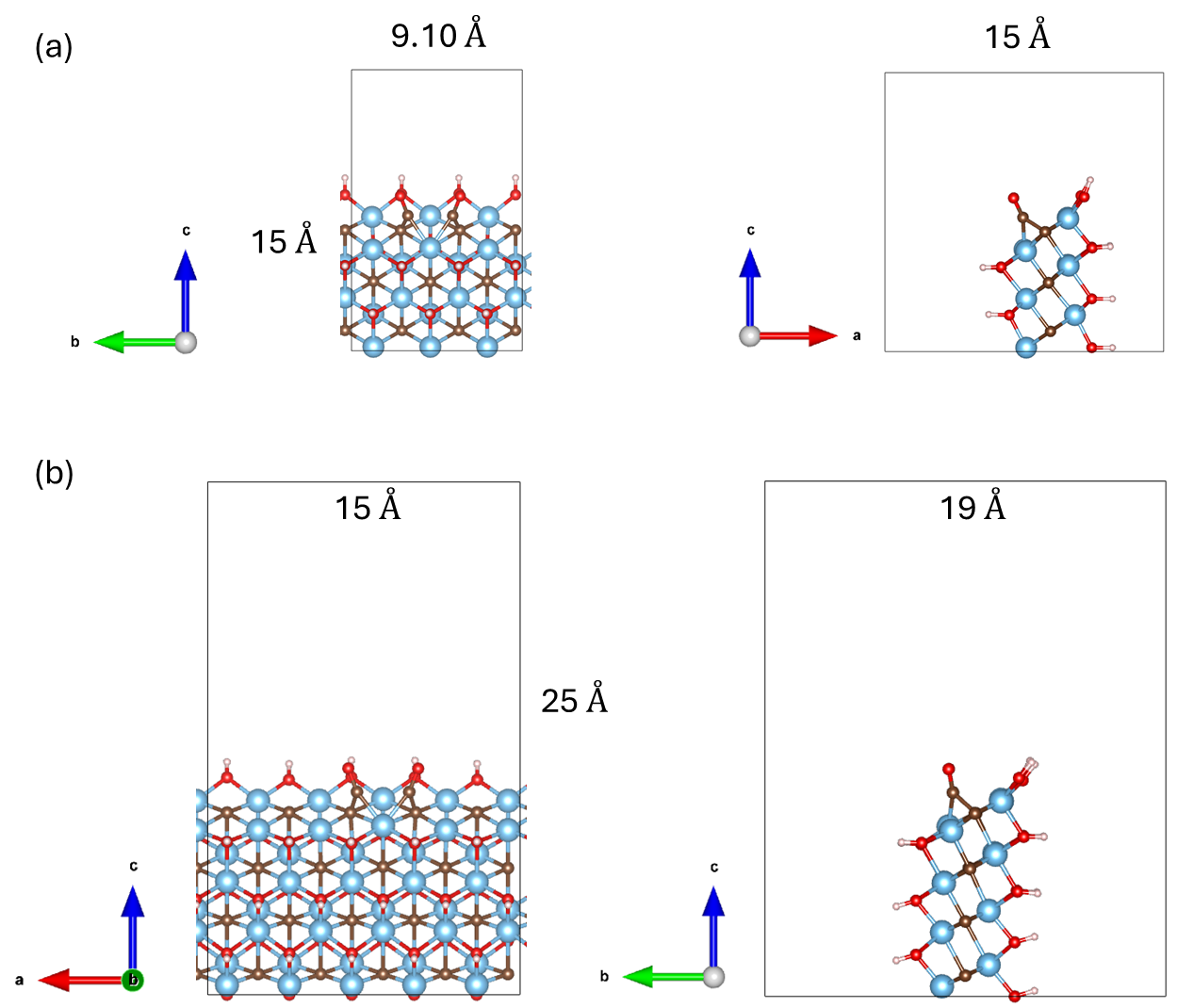


Fig. S15 (a) Schematic of the simulation supercell (15 × 9.10 × 15 Å³). (b) Schematic of the larger supercell (15 × 19 × 25 Å³) used for convergence tests.


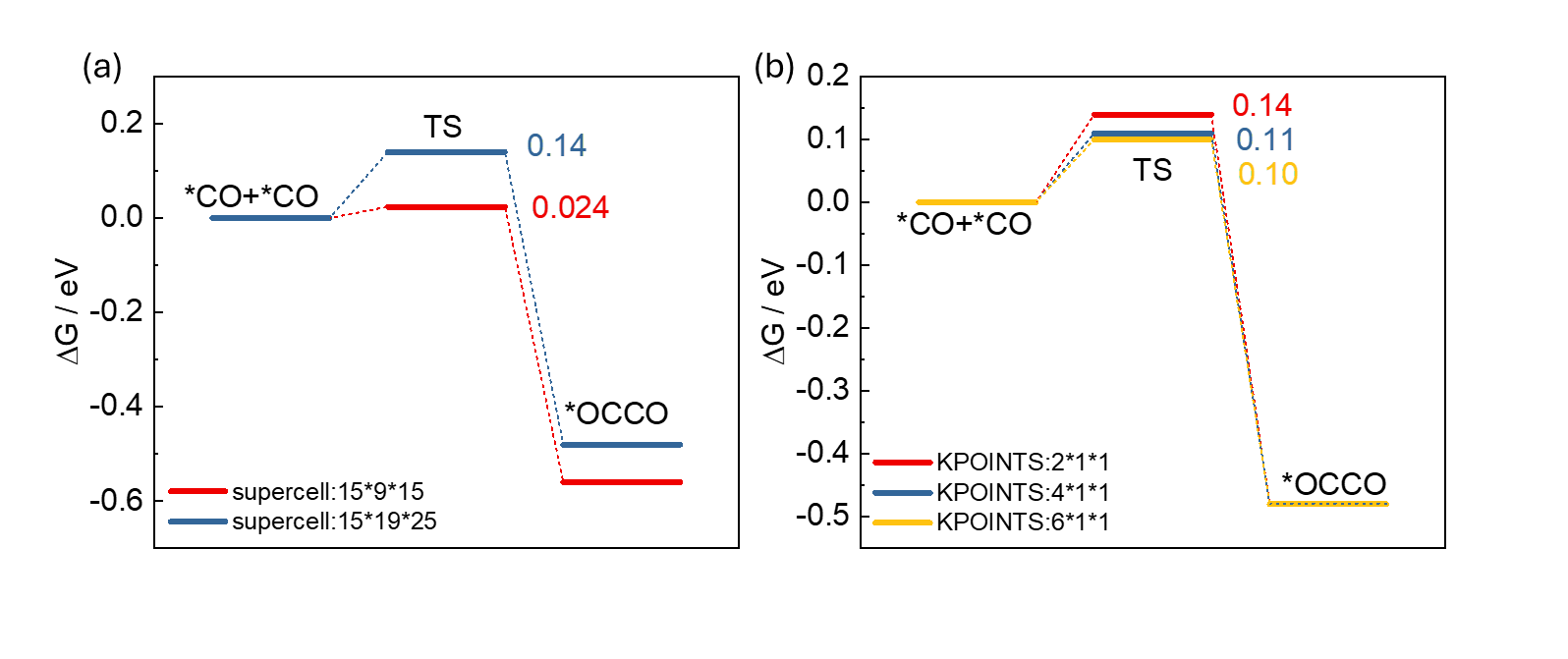


Fig. S16 (a) Convergence test in terms of supercell size: C-C coupling barrier after expansion. (b) Convergence test in terms of k-point density: C-C coupling barrier with a refined k-mesh.


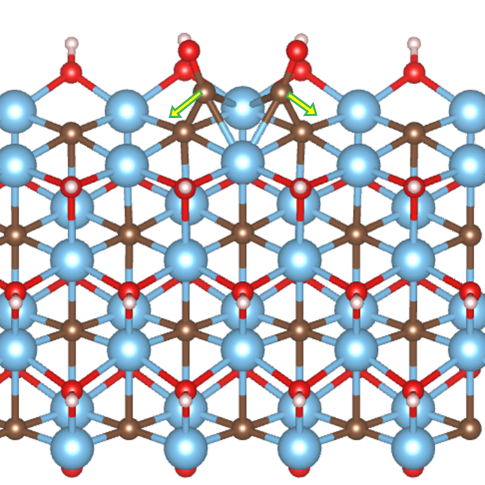


Fig. S17 Vibrational mode corresponding to the imaginary frequency of carbon atoms in the *CO dimerization transition states on the ZZ3* of Ti_2_C(OH)_2_.


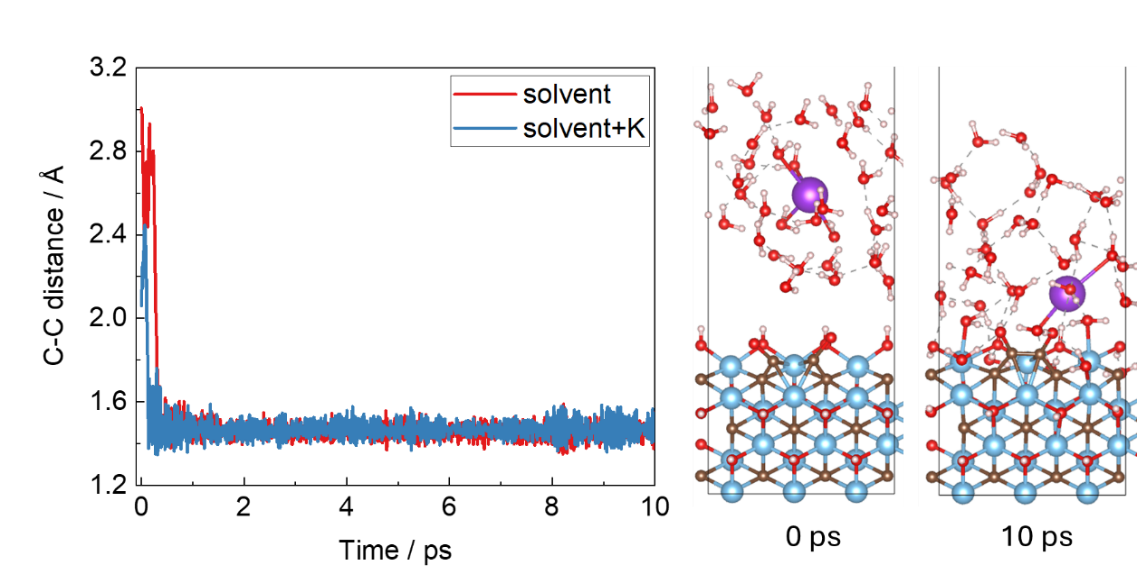


Fig. S18 Evolution of C-C bond distance during AIMD simulation in solvent environment with and without one K^+^.


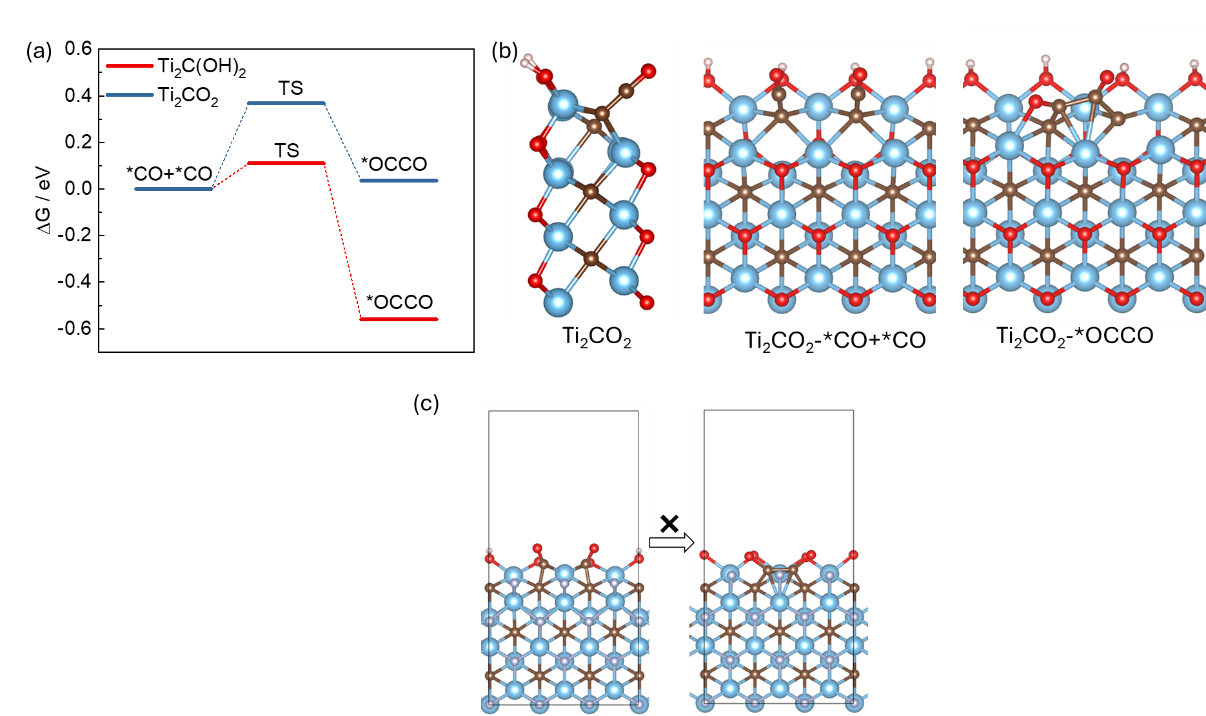


Fig. S19 (a) Reaction pathway and energy barrier for CO dimerization on Ti_2_CO_2_ ZZ3*.
(b) Corresponding structural evolution from adsorbed *CO to the coupled *OCCO intermediate. (c) Under the convergence criterion, CO cannot be coupled together on Ti_2_CF_2_ ZZ3.


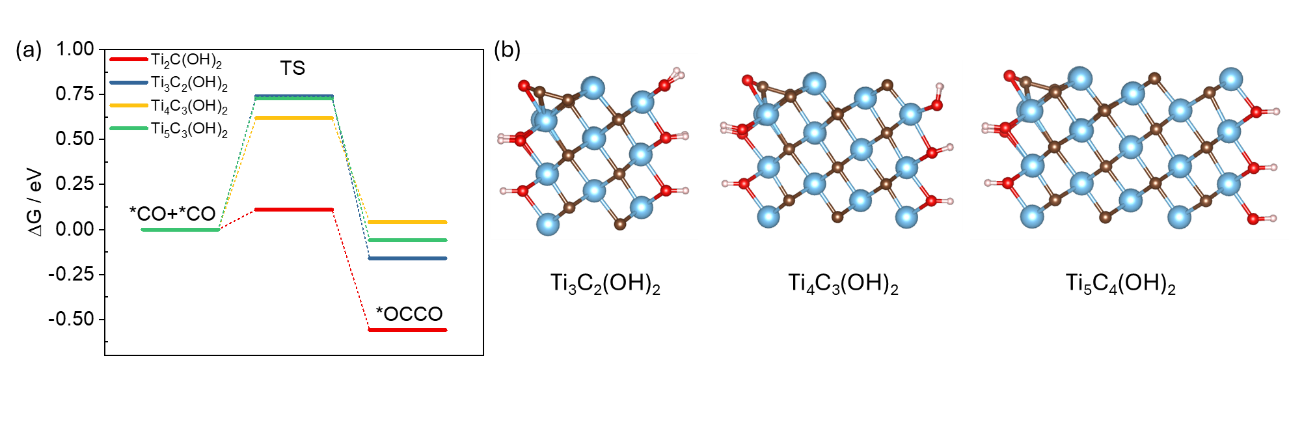


Fig. S20 (a) Reaction pathway and energy barrier for CO dimerization on ZZ3 of Ti_3_C_2_(OH)_2_, Ti_4_C_3_(OH)_2_ and Ti_5_C_4_(OH)_2_. (b) Schematic diagram of Ti-based MXenes of different thicknesses.


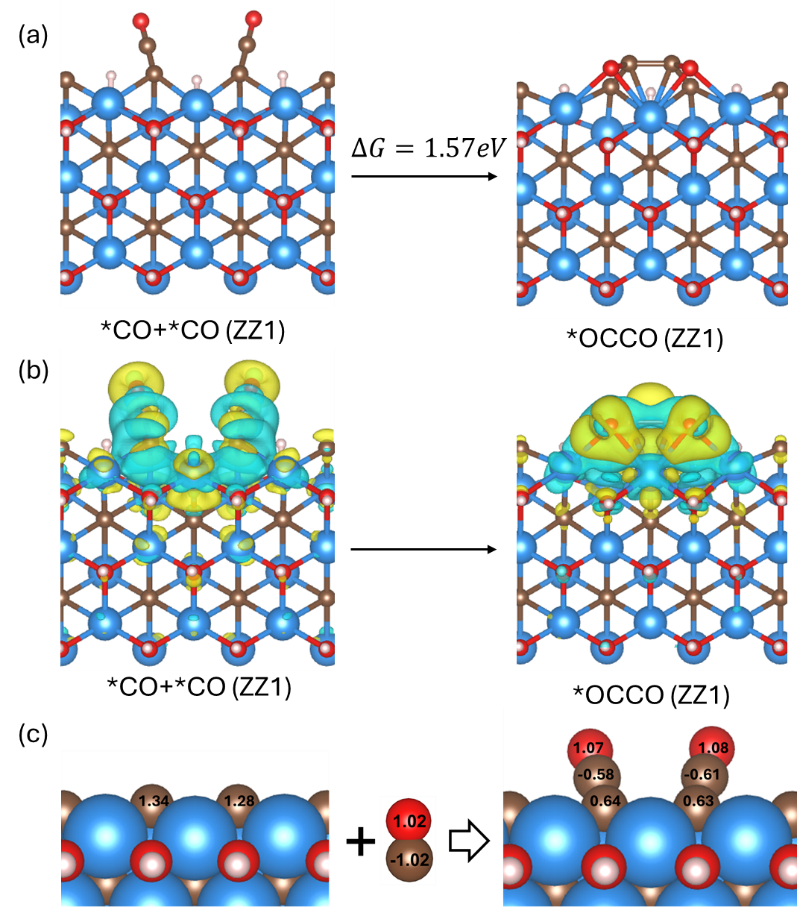


Fig. S21 (a) Adsorption configurations of dual *CO adsorbates on Ti_2_C(OH)_2_ ZZ1 and the resulting *OCCO complex after C–C coupling, along with the calculated Gibbs free energy change for this step. (b) Differential charge density in the *CO-dimerization process on the ZZ1 of Ti_2_C(OH)_2_ (isosurface = ±0.003 eÅ⁻³). (c) Bader charge evolution during the CO adsorption on the ZZ1 of Ti_2_C(OH)_2_ (positive/negative value indicates electron gain/loss).


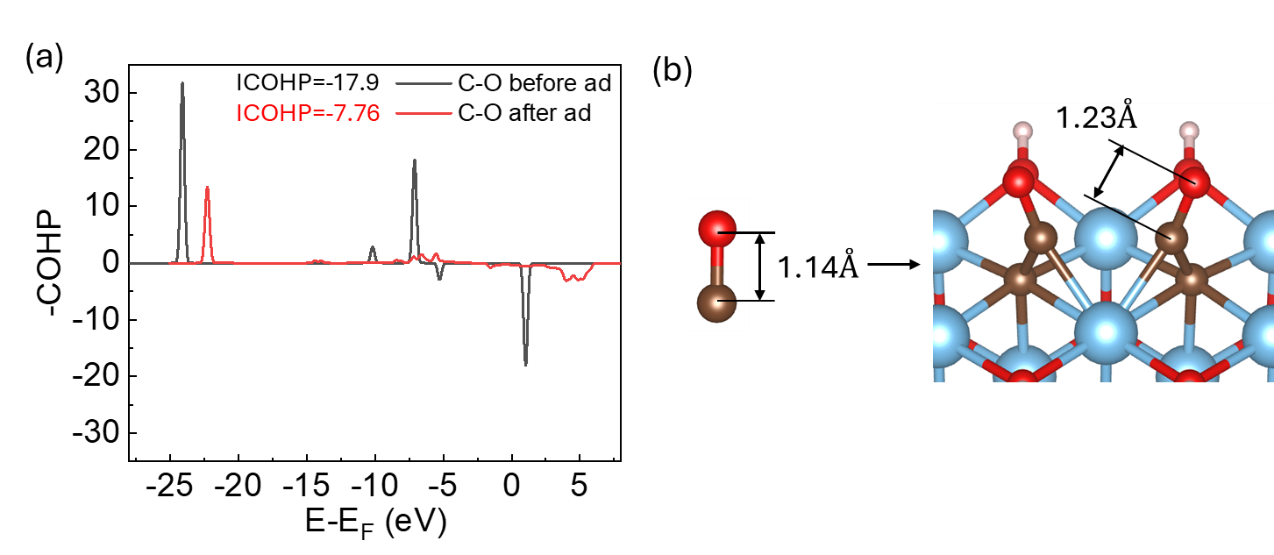


Fig. S22 (a) Projected crystal orbital Hamiton population (pCOHP) analysis for CO before and after adsorption. (b) The bond length of CO before and after adsorption.


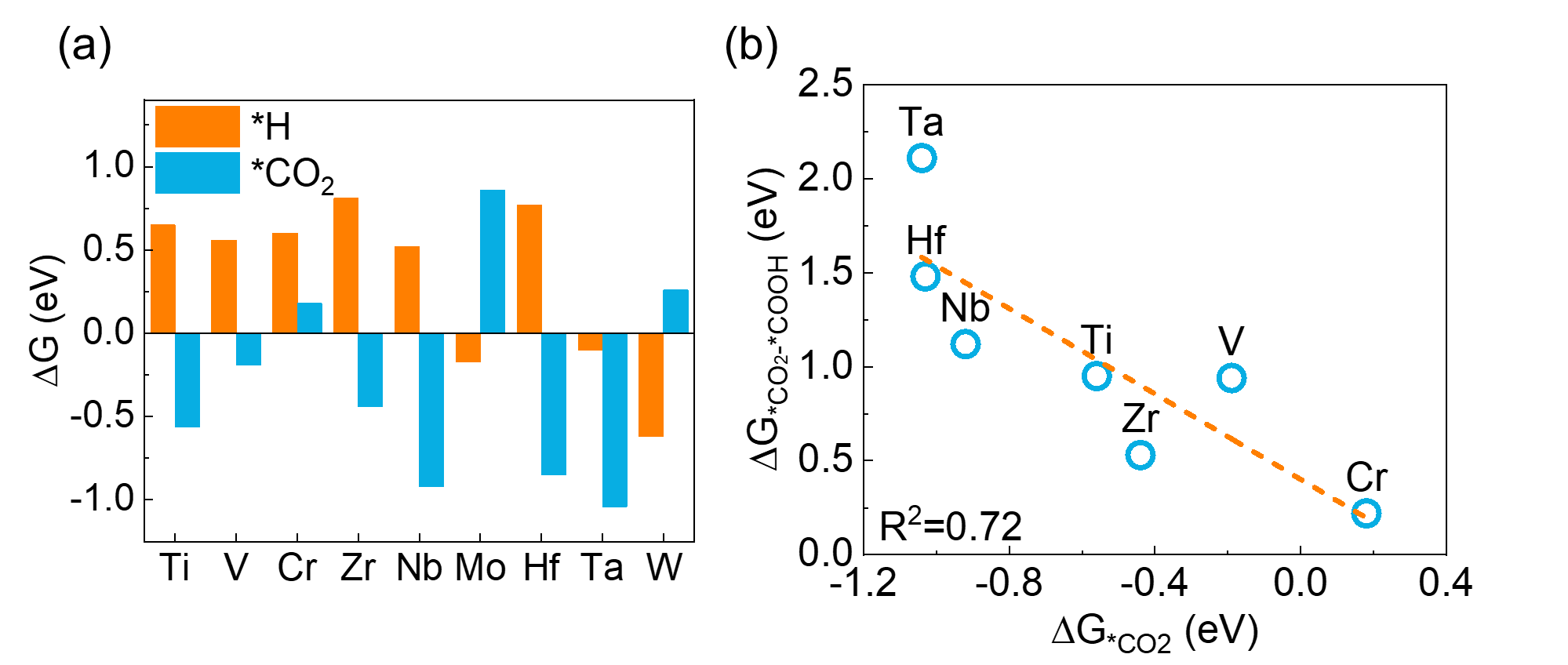


Fig. S23 (a) H vs CO_2_ adsorption at ZZ3* of Ti_2_C(OH)_2_. (b) Correlation: CO_2_ adsorption energy vs $\Delta G$ for CO_2_-COOH.


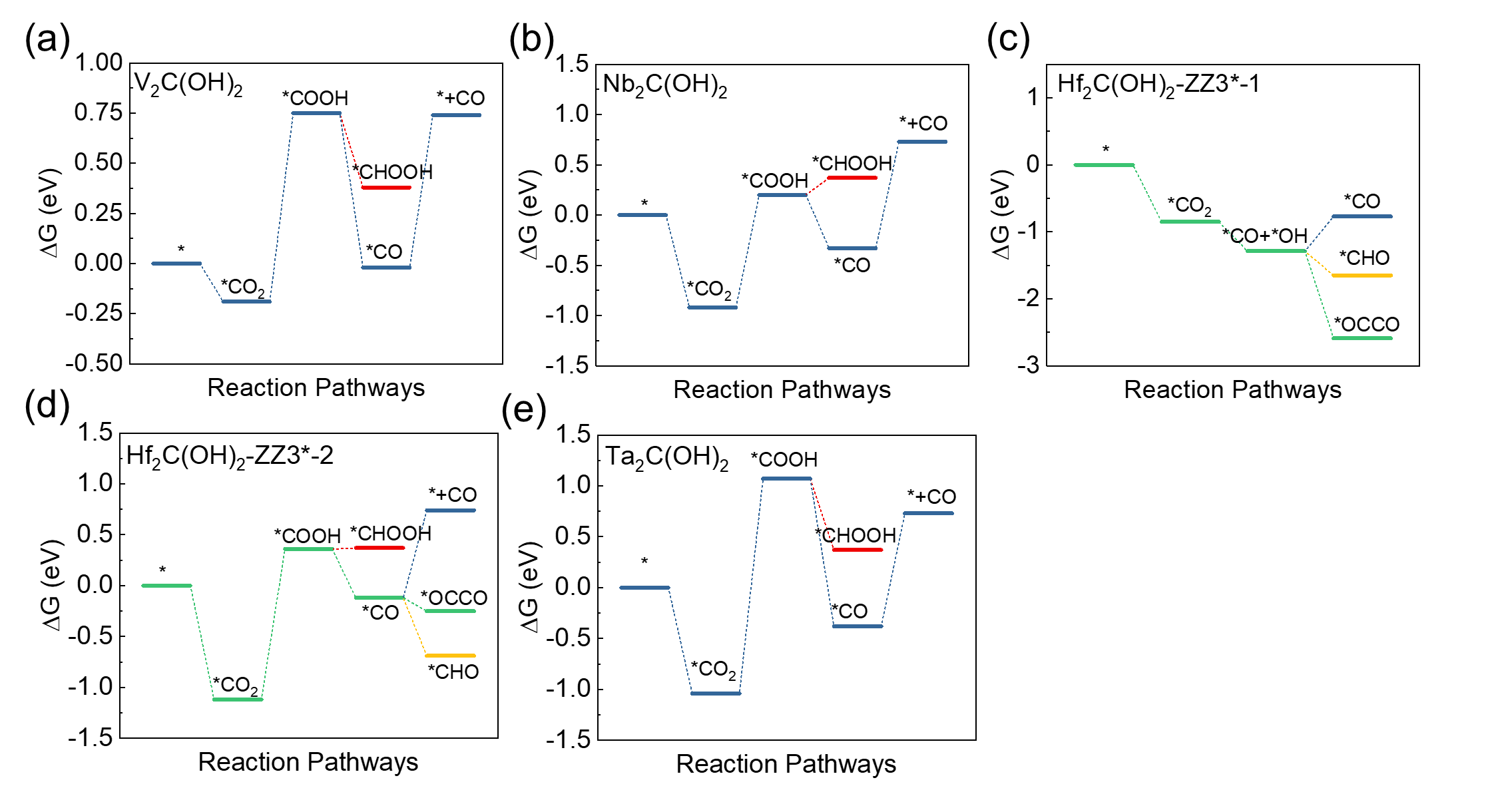


Fig. S24 Free energy diagram of CO_2_ reduction pathways on the ZZ3* of (a) V, (b) Nb, (c)&(d)Hf and (e) Ta-based MXenes. Note: There are two structures of ZZ3* of Hf_2_C(OH)_2_, as shown in Fig. S8b. The dissociation of *COOH on the Hf_2_C(OH)_2_-ZZ3*-1 shows the strong bonding between CO_2_ and Hf sites and the hydrogenation of CO_2_ can refer to Hf_2_C(OH)_2_-ZZ3*-2 (Fig. S23d).


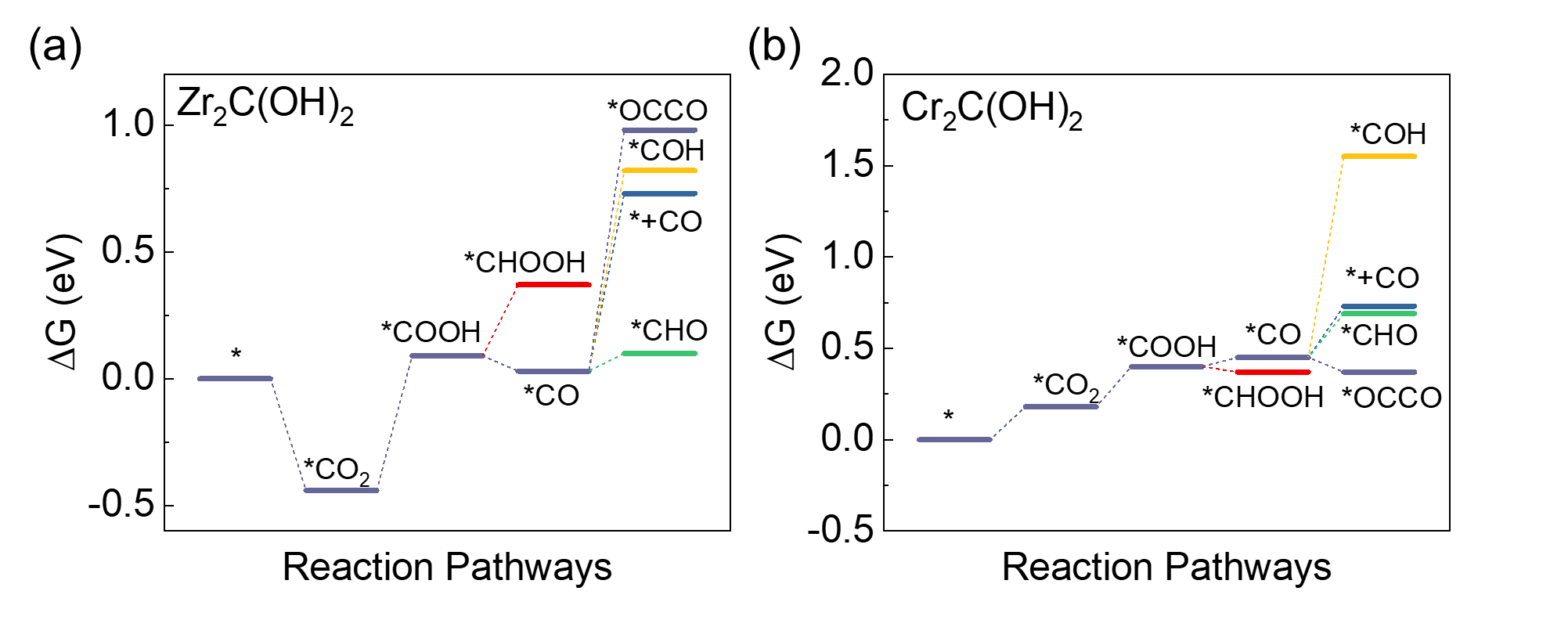


Fig. S25 Free energy diagram of CO_2_ reduction pathways on the ZZ3* of (a) Zr and (b) Cr-based MXenes.


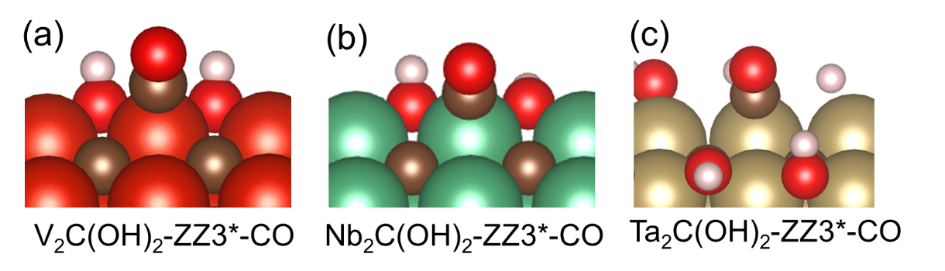


Fig. S26 CO adsorption configurations on ZZ3* of (a) V_2_C(OH)_2_, (b) Nb_2_C(OH)_2_ and (c) Ta_2_C(OH)_2_.


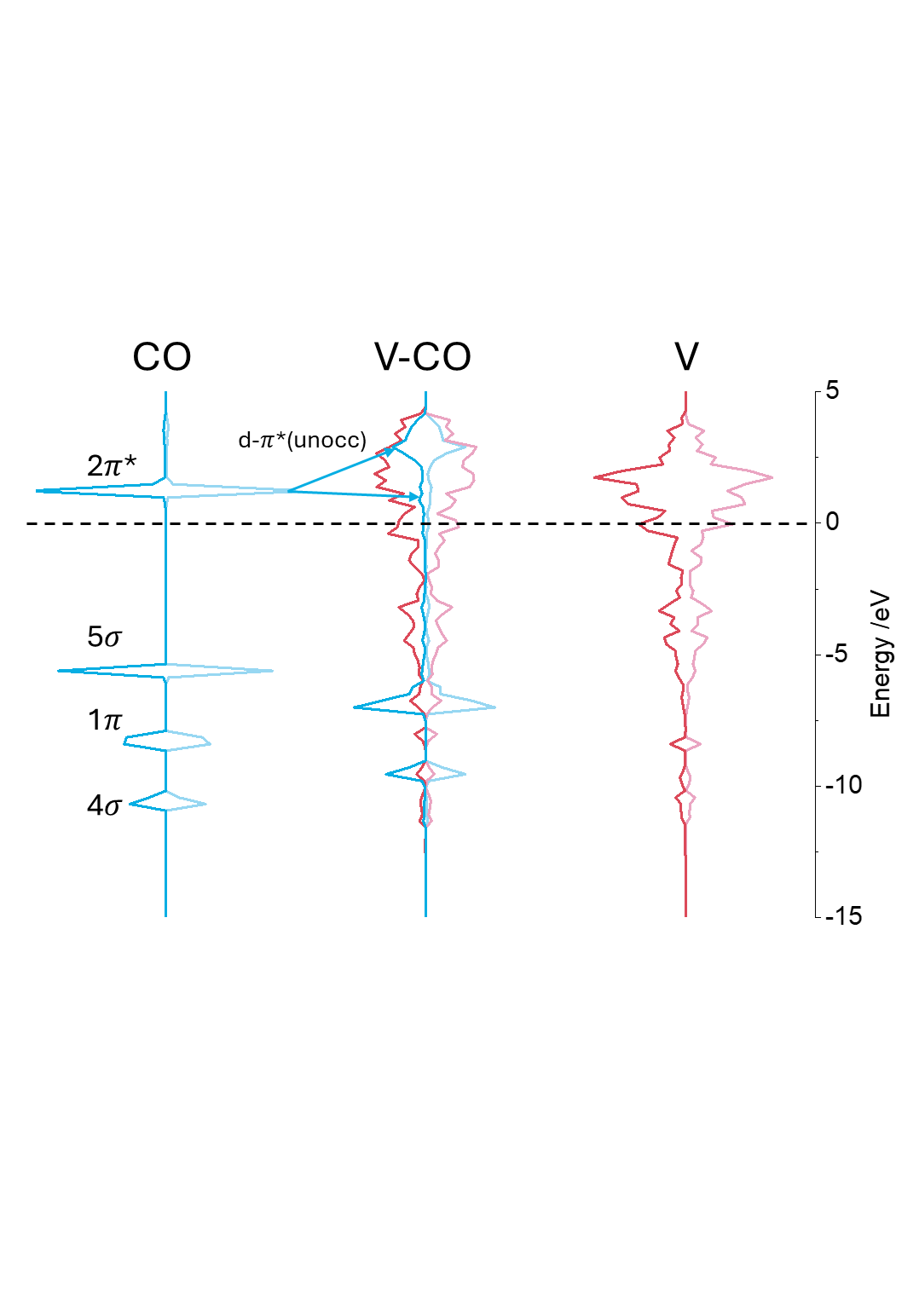


Fig. S27 pDOSs of CO gas molecule, V_2_C(OH)_2_-ZZ3*-CO and V atom (adsorption site).


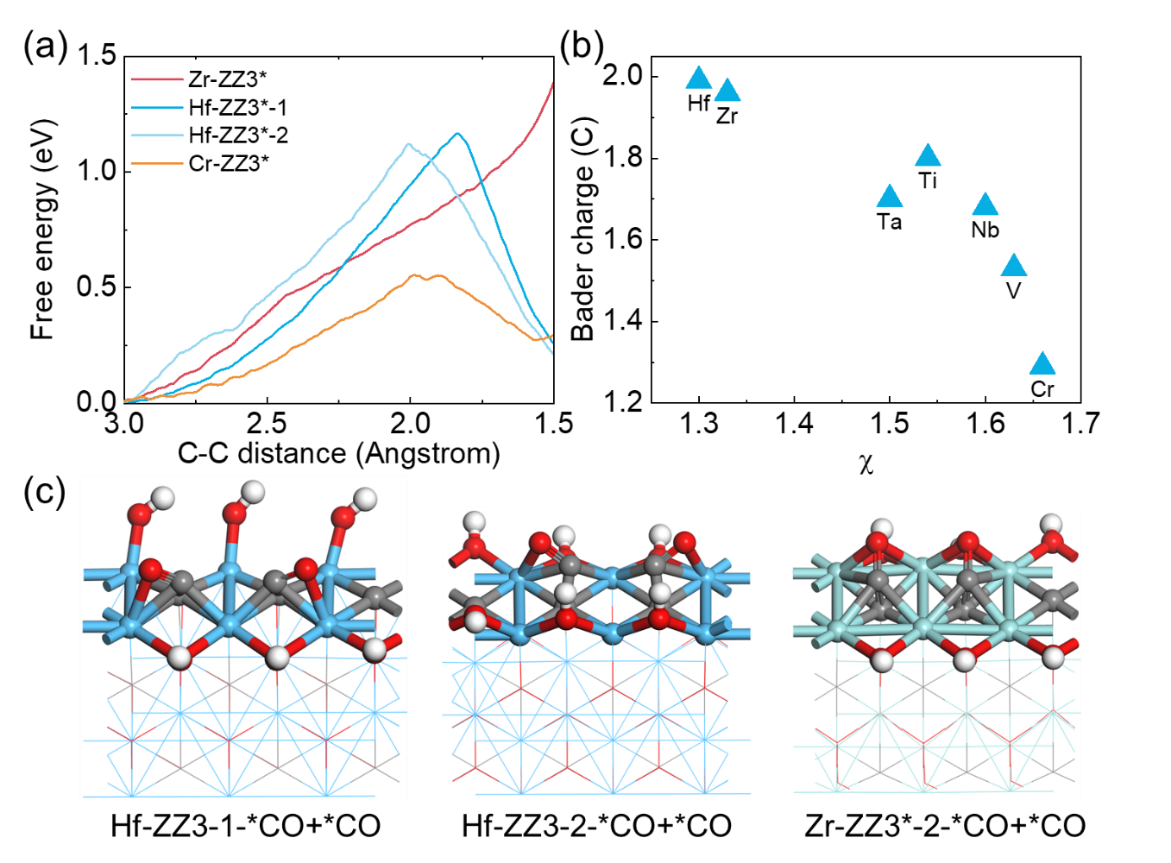


Fig. S28 (a) Comparative free energy profiles for *CO-*CO coupling reactions on different MXene ZZ3*. (b) Correlation between Bader charge accumulation at *C_lat_ sites and the Pauling electronegativity of constituent transition metals. (c) Optimized *CO adsorption geometries on Hf_2_C(OH)_2_ and Zr_2_C(OH)_2_ ZZ3* edges.


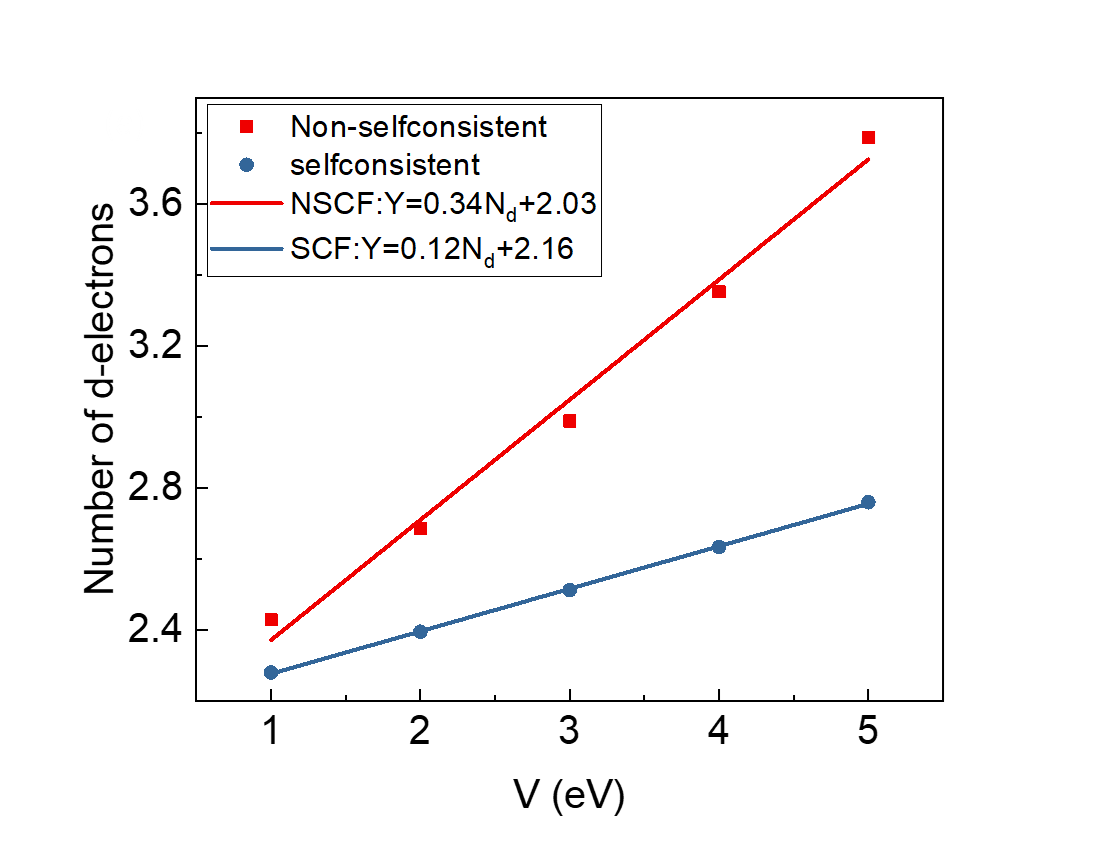


Fig. S29 Calculated d-band electron population of Ti atoms across different U values obtained from linear response calculations in Ti_2_C(OH)_2_.^1^ The U value can be obtained by using the slope obtained from linear fitting according to the formula: U=$\chi^{-1}-\chi_{0}^{-1}\approx\left( \frac{\partial N_{I}^{SCF}}{\partial V_{I}} \right)^{-1}-\left( \frac{\partial N_{I}^{NSCF}}{\partial V_{I}} \right)^{-1}$. The U values ​​of metal atoms in other MXenes obtained by the linear response method are listed in Table S3.


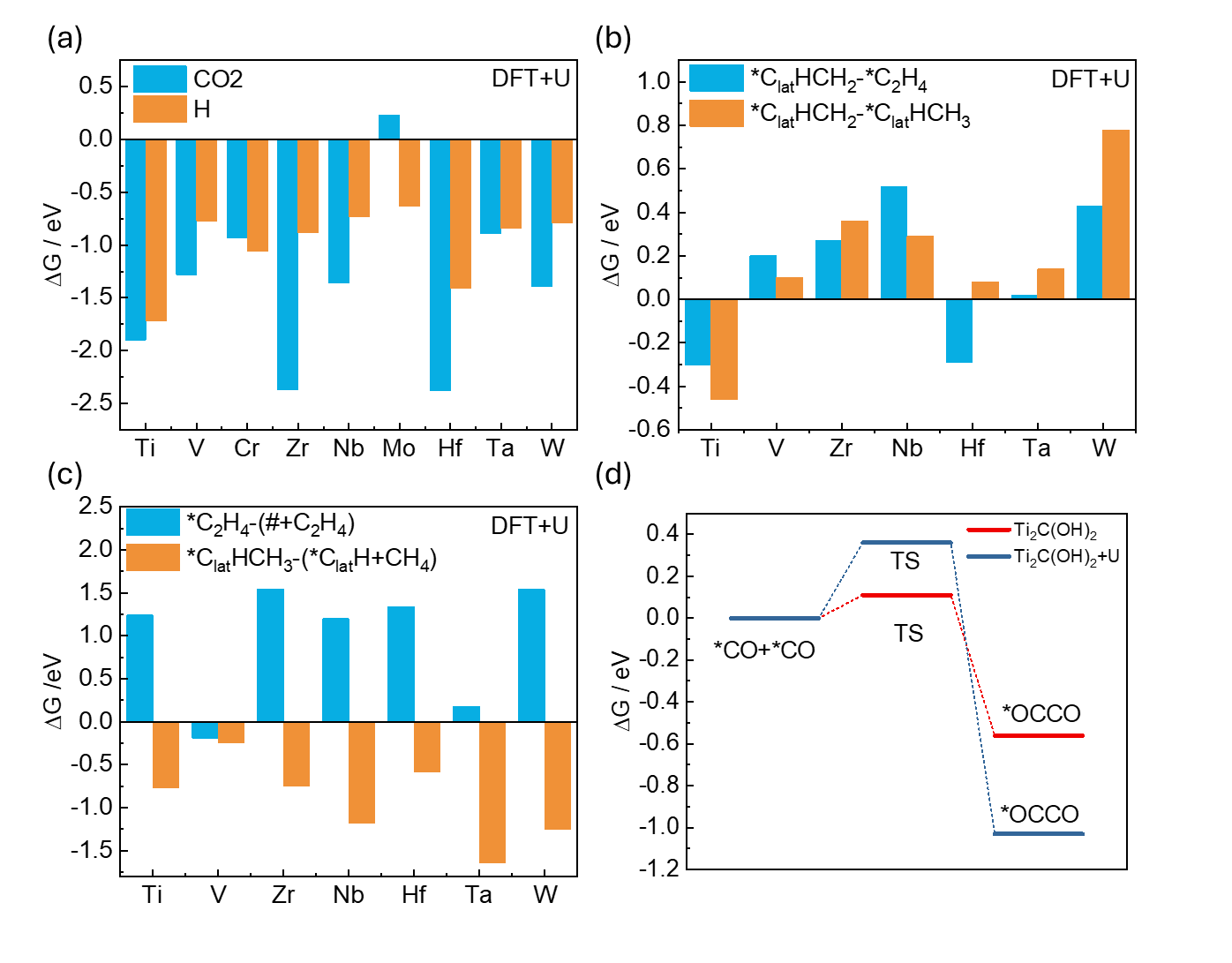


Fig. S30 (a) Adsorption energies of H and CO_2_ on the ZZ1 of various metal carbides under the +U correction. (b) Gibbs free energy changes under +U for the hydrogenation of *C_lat_HCH_2_ to *C_lat_HCH_3_ (C_1_ path) or *C_2_H_4_ (C_2_ path). (c) Comparison of free energies under +U for the C_2_H_4_ desorption and CH_4_ formation steps. (d) Energy barrier for CO coupling with the +U correction applied.

Table. S1 Total energies (eV) of M_2_C(OH)_2_ unit cells with different OH functionalization configurations.

|  | Fcc-type | Hcp-type | Asymmetric |
| --- | --- | --- | --- |
| Ti_2_C(OH)_2_ | **-52.27** | -51.94 | -52.15 |
| V_2_C(OH)_2_ | **-52.17** | -51.99 | -51.96 |
| Cr_2_C(OH)_2_ | -50.01 | -49.91 | **-50.03** |
| Zr_2_C(OH)_2_ | **-54.24** | -52.98 | -53.77 |
| Nb_2_C(OH)_2_ | **-54.91** | -54.71 | -54.60 |
| Mo_2_C(OH)_2_ | -52.52 | **-52.71** | -52.58 |
| Hf_2_C(OH)_2_ | -55.85 | -56.34 | **-56.46** |
| Ta_2_C(OH)_2_ | -57.76 | **-57.94** | -57.67 |
| W_2_C(OH)_2_ | -56.07 | **-56.43** | -55.94 |

Table S2. The vibrational frequencies of the C atoms in the transition states of *CO dimerization on ZZ3* of Ti_2_C(OH)_2_.

| Vibration modes of the C atoms | Frequencies / cm^-1^ |
| --- | --- |
| 1 f | 1571.517 |
| 2 f | 1535.339 |
| 3 f | 630.2494 |
| 4 f | 497.9335 |
| 5 f | 426.5542 |
| 6 f | 406.9508 |
| 7 f | 341.5975 |
| 8 f | 245.8754 |
| 9 f | 188.4536 |
| 10 f | 140.5132 |
| 11 f | 58.13374 |
| 12 f/i | 150.153 |

Table S3. DFT+U parameters (U) determined by the linear response method.

|  | Slope (NSCF) | Slope (SCF) | U |
| --- | --- | --- | --- |
| Ti | 0.34 | 0.12 | 5.39 |
| V | 0.46 | 0.14 | 4.97 |
| Cr | 0.51 | 0.15 | 4.71 |
| Zr | 0.30 | 0.16 | 2.92 |
| Nb | 0.40 | 0.29 | 0.95 |
| Mo | 0.50 | 0.21 | 2.76 |
| Hf | 0.23 | 0.13 | 3.34 |
| Ta | 0.33 | 0.16 | 3.22 |
| W | 0.36 | 0.17 | 3.10 |

#Reference

1. M. Cococcioni and S. de Gironcoli, Linear response approach to the calculation of the effective interaction parameters in theLDA+Umethod, Physical Review B 2005, 71, 035105.
